# Cilia defects upon loss of WDR4 are linked to proteasomal hyperactivity and ubiquitin shortage

**DOI:** 10.1038/s41419-024-07042-5

**Published:** 2024-09-09

**Authors:** Martin D. Burkhalter, Tom Stiff, Lars D. Maerz, Teresa Casar Tena, Heike Wiese, Julian Gerhards, Steffen A. Sailer, Linh Anna Trúc Vu, Max Duong Phu, Cornelia Donow, Marius Alupei, Sebastian Iben, Marco Groth, Sebastian Wiese, Joseph A. Church, Penelope A. Jeggo, Melanie Philipp

**Affiliations:** 1 Department of Experimental and Clinical Pharmacology and Pharmacogenomics, Section of Pharmacogenomics, Eberhard-Karls-University Tübingen, 72074 Tübingen, Germany; 2 Genome Damage and Stability Centre, University of Sussex, Brighton, BN1 9RQ UK; 3 Institute for Biochemistry and Molecular Biology, Ulm University, 89081 Ulm, Germany; 4 Core Unit Mass Spectrometry and Proteomics, Medical Faculty, Ulm University, 89081 Ulm, Germany; 5 Department of Dermatology, Ulm University, 89081 Ulm, Germany; 6 Core Facility Next Generation Sequencing, Leibniz Institute on Aging–Fritz Lipmann Institute, 07745 Jena, Germany; 7 Department of Pediatrics, Keck School of Medicine of University of Southern California, Children’s Hospital Los Angeles, Los Angeles, CA 90033 USA

**Keywords:** Ciliogenesis, Disease model

## Abstract

The WD repeat-containing protein 4 (WDR4) has repeatedly been associated with primary microcephaly, a condition of impaired brain and skull growth. Often, faulty centrosomes cause microcephaly, yet aberrant cilia may also be involved. Here, we show using a combination of approaches in human fibroblasts, zebrafish embryos and patient-derived cells that WDR4 facilitates cilium formation. Molecularly, we associated WDR4 loss-of-function with increased protein synthesis and concomitant upregulation of proteasomal activity, while ubiquitin precursor pools are reduced. Inhibition of proteasomal activity as well as supplementation with free ubiquitin restored normal ciliogenesis. Proteasome inhibition ameliorated microcephaly phenotypes. Thus, we propose that WDR4 loss-of-function impairs head growth and neurogenesis via aberrant cilia formation, initially caused by disturbed protein and ubiquitin homeostasis.

## Introduction

Primary microcephaly (PM) is a failure in the growth of the brain and skull, which can be diagnosed from 32 weeks of gestation using ultrasound. Microcephaly is defined by reduced head circumference by at least 2 standard deviations below the median. Such reduction in head circumference directly correlates with a smaller size of an underdeveloped brain. As a consequence, infants with PM suffer from a number of symptoms including cognitive and motoric impairments, epilepsy, cerebral palsy, a delay in speaking and defects of the eyes [1]. Some PM children also show overall growth reduction such as in the case of Seckel or Meier-Gorlin Syndromes, which is then called microcephalic primordial dwarfism [2].

Despite the rare occurrence of the condition, several genetic loci have been linked to PM. We and others have shown that proteins required for DNA replication (i.e., ORC1), DNA damage response (i.e., ATR, ATRIP) and DNA repair (i.e., PRKDC, XRCC4, DNA ligase IV) allow for faithful brain growth [3–10]. Interestingly, in addition to their canonical function, namely maintenance of intact DNA and cell division, the formation of aberrant centrosomes can be observed upon loss of ORC1 or ATR [11, 12]. In addition, centrosomal aberrations can provoke PM, too, through reduced self-renewal and premature differentiation of neuronal progenitors, altered duration of the cell cycle, as well as a delay in mitosis [13]. Centrosomes, however, provide also the basal bodies for cilia, which are crucial cell organelles involved in cellular signal sensing, signal transduction and cell cycle progression [14]. Thus, microcephaly may arise also in the context of impaired cilia formation or function and therefore could be a disorder with ciliary contribution [11, 12, 15]. Additionally, genes with as yet unknown or less investigated functions have been identified as drivers of microcephaly when mutated. One such gene is *WDR4*, which encodes for WD repeat-containing protein 4 (also known as TRM82, tRNA methyltransferase 82 homolog or protein Wuho homolog) [16]. WDR4 is a WD40 domain containing protein, which is best known for its interaction with the methyltransferase METTL1 [17–19]. WDR4 promotes activity of METTL1 to generate N7-methylguanosine (m^7^G) tRNAs at position 46 to stabilize tRNAs, that has been shown to be of importance for maintenance and differentiation of embryonic stem cells [20]. The Drosophila homolog to WDR4, Wuho, has been linked to impaired fertility defects, perhaps due to impaired genomic stability [21]. Increased levels of WDR4 and its interaction partner METTL1 have been observed in various tumors suggesting that these two factors together increase cancer susceptibility [22–24]. Mechanistically, METTL1/WDR4 have been connected to different pathways to increase carcinogenesis, such as the WNT pathway as well as autophagy [25, 26]. However, Wang and colleagues elegantly showed that overexpression of WDR4 alone in A459 cells is sufficient to induce metastatic nodules in the lung upon transplantation of these cells [27]. In this case, WDR4 works as a substrate adapter of the Cullin 4 E3 ubiquitin ligase complex, which specifically targets the tumor suppressor promyelocytic leukemia (PML) for degradation [27].

Genetic variations of *WDR4* have been repeatedly reported in the context of microcephalic disorders such as Galloway-Mowat syndrome 6 (OMIM 618347) and microcephaly, growth deficiency seizures and brain malformations (OMIM 618346). Several genetic variants have been identified in affected individuals all over the world suggesting that WDR4 is an essential protein for normal brain and skull development [28–31]. Indeed, the Chen lab recently showed in mice that WDR4 supports development of the cerebellum by activating the small GTPase RAC1 through degradation of Arhgap17, a negative regulator of RAC1 [32]. This prevents premature cell cycle exit and differentiation of progenitor cells needed for cerebellum development [32]. However, since PM induced by WDR4 dysfunction affects the whole brain, we wondered whether other mechanisms contribute to the phenotypes observed in patients [28–31]. We here show that WDR4 is needed to properly generate cilia, organelles important for cell signaling and brain development. Our data suggest that this function of WDR4 can be separated from its canonical interaction partner METTL1. Instead, dysregulated protein homeostasis involving changing ubiquitin distribution presents as the most likely driver for disturbed ciliogenesis in absence of WDR4.

## Results

**Fig. 1:**
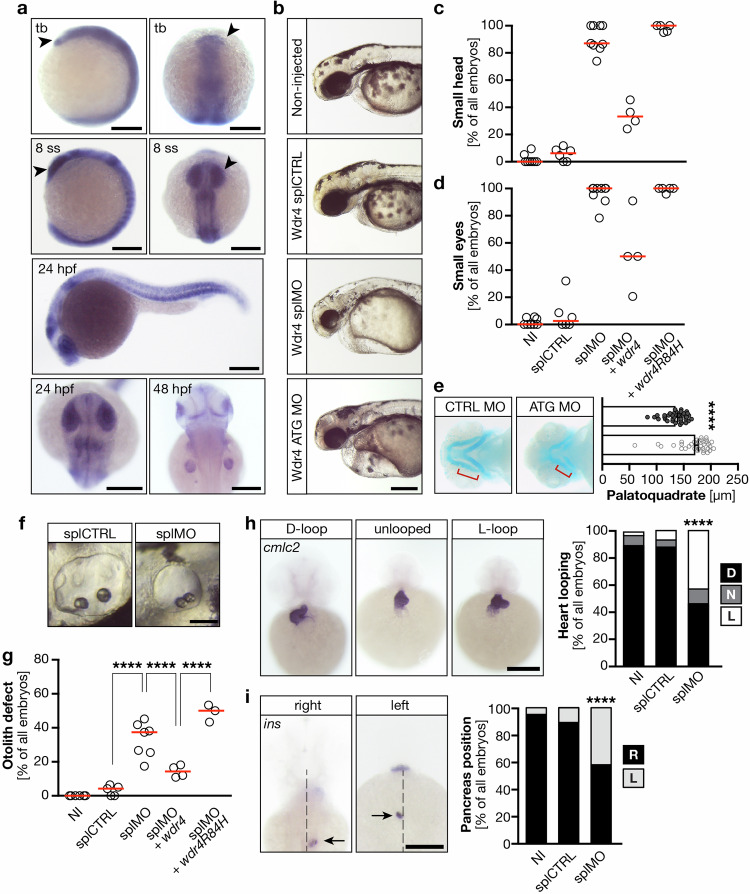
Wdr4 is expressed in and required for the developing brain. **a** Whole mount in situ hybridization (WMISH) revealed expression of *wdr4* in anterior structures of the zebrafish embryo (arrow heads) including the developing brain. Tb tailbud stage; 8 ss 8 somite stage; hpf hours post fertilization. Scale bars: 200 µm. **b** Live images of 48 hpf zebrafish embryos, which were either non-injected (NI) or injected with one of the following MOs: splCTRL, splMO or a MO blocking translation of Wdr4 (Wdr4 ATG MO). Scale bar: 250 µm. **c** Scatter plot showing the incidence of microcephaly in zebrafish upon Wdr4 KD. Co-injection of capped RNA encoding zebrafish Wdr4 rescues head size, while the patient-resembling Wdr4 R84H does not. *n* = 3–9 experiments with 93–244 embryos in total. **d** Loss of Wdr4 results in zebrafish embryos with smaller eyes, which cannot be fully rescued by *wdr4 R84H* injection. *n* = 3–9 experiments with 93–244 embryos in total. **e** Reduced size of cartilage structures in the developing head as shown by Alcian blue staining. Graph shows measurement of the palatoquadrate (red bracket). Lower bar shows control. *n* = 16/15; ****p < 0.0001, Mann–Whitney test. **f** Images of the otoliths in the otic vesicle upon injection of splCTRL or Wdr4 splMO. Scale bar: 10 µm. **g** Knockdown of Wdr4 increases the percentage of embryos with fused otoliths. *n* = 3–9 experiments with 93–244 embryos in total. ****p < 0.0001. One-way ANOVA with Sidak’s multiple comparison test. **h** Loss of Wdr4 randomizes heart looping in 48 hpf zebrafish embryos. D, correct D-loop; N, no loop; L, inverse loop. *n* = 4 experiments with 63–94 embryos. ****p < 0.0001. Fisher’s exact test. Scale bar: 200 µm. **i** Pancreas placement is randomized upon Wdr4 KD. R, pancreas right from the midline (dotted line); L, pancreas left from midline. Arrows indicate *insulin* *n* = 4 experiments with 63–94 embryos in total. ****p < 0.0001. Fisher’s exact test. Scale bar: 200 µm. Red lines: median.

Despite WDR4 having been associated to PM, the mechanism how this factor affects embryonal development so far has not been completely understood. Because of its many advantages including rapid development of translucent embryos as well as high conservation to mammalian species we turned to zebrafish as model organism, in which a homolog of human *WDR4* exists. In situ hybridization experiments showed that *wdr4* is expressed throughout development with enrichment in neural structures such as the developing eyes, brain and neural tube (Fig. 1a). Consistent with reports in patients and mice [28–32] and the expression pattern in zebrafish, we found that depletion of Wdr4 using an antisense morpholino oligonucleotide (MO)-mediated approach resulted in zebrafish with smaller heads and eyes (Figs. 1b–d and S1a, b). Two different MOs were tested, one interfering with translation of *wdr4* mRNA into protein and one, which prevents regular splicing (Fig. S2). Both MOs provoked similar defects in the formation of anterior structures. To further characterize this defect, we performed Alcian blue staining of 4 days post fertilization (4 dpf) embryos and found that formation of cartilaginous structures was still occurring, however to a lesser extent with structures appearing shortened compared to controls (Fig. 1e). This is consistent with typical PM phenotypes.

In our quest for understanding the underpinnings of WDR4-associated PM better, we performed rescue experiments. To this end, we co-injected capped RNA encoding either wild-type Wdr4 or one encoding a mutant version, which mimics PM patients with a mutation in *WDR4*. Cells of these patients have been archived at the cell repository of the University of Sussex as the patients displayed a Seckel syndrome-like phenotype. The patients carry a variant of *WDR4*, which results in the exchange of arginine at position 84 with histidine (*wdr4 R84H*) leading to reduced expression of WDR4 (Fig. S3). This residue is conserved in zebrafish *wdr4*. Reconstitution of Wdr4 knockdown (KD) embryos with wild-type *wdr4* at least partially restored growth of anterior structures (Fig. 1c, d) suggesting that the phenotypes after Wdr4 MO injection are specific. The same approach using *wdr4 R84H*, however, was unable to rescue head and eye size in zebrafish embryos (Fig. 1c, d), while injection of the RNAs alone had no effect (Fig. S1c, d). These results link the phenotypes in zebrafish embryos to PM in patients carrying genetic variants of WDR4.

Closer examination of Wdr4 KD embryos revealed additional phenotypes such as defects in otolith seeding (Figs. 1f, g and S1e, f). Otolith formation and positioning is facilitated through concerted beating of cilia within the otic vesicle resulting in the accumulation of calcium carbonate crystals on particular matrix proteins [33]. In case of cilia defects, the number of otoliths may be changed or, as observed here, otoliths become symmetrical and centered in the lower half of the otic placode (Fig. 1f). Reconstitution with wild-type Wdr4 significantly rescued the otolith defect, while the patient variant failed to do so (Fig. 1g). To test whether loss of Wdr4 causes also other defects in cilium function, we analyzed the situs of the injected embryos The situs of inner organs is determined early during embryogenesis through the development of an internal left-right asymmetry along the anterior-posterior axis. This is mediated by cilia in a temporal organ of laterality, which is called Kupffer’s vesicle (KV) in zebrafish [14]. When cilia do not form properly or become dysfunctional in the KV, left-right asymmetry will not be established properly and organs may end up on the wrong side of the body. Experimentally, this can be scored by heart looping direction and placement of abdominal organs. Wdr4 KD indeed produced more often embryos with inversely looped or even unlooped hearts than controls (Figs. 1h and S1g). Similarly, Wdr4 KD also randomized pancreas placement as observed by the expression of *insulin*, which marks the primary endocrine islet (Figs. 1i and S1h). These results suggest an unanticipated function of WDR4 in cilium biology.

**Fig. 2:**
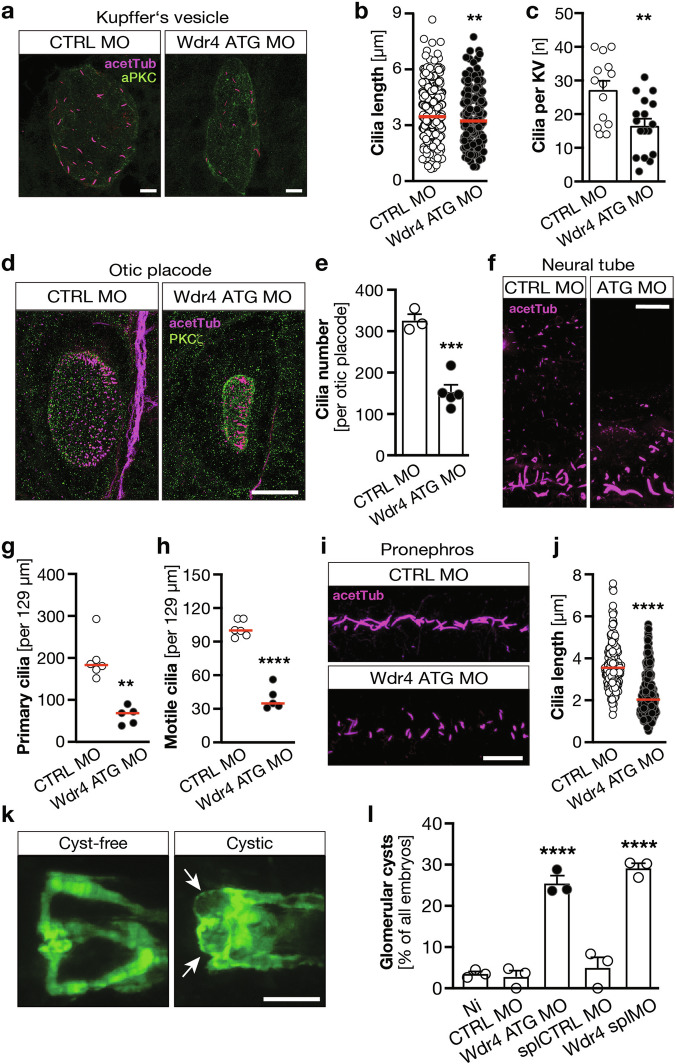
Lack of Wdr4 affects cilia in multiple organs in zebrafish. **a** Representative images of cilia in the Kupffer’s vesicle (KV) of 8 ss zebrafish embryos. Cilia in magenta (acetylated tubulin), green counterstain (PKCz) to visualize the KV outline. Scale bar: 10 µm. **b** KV cilia are shorter in the absence of Wdr4. *n* = 2 experiments with 14–17 KVs and 283–379 cilia in total. **p = 0.0066. Mann–Whitney test. **c** Zebrafish embryos depleted of Wdr4 extend fewer cilia into the KV. *n* = 2 experiments with 14–17 KVs in total. **p = 0.0034. Welch’s *t* test. Mean ± SEM. **d** Loss of Wdr4 reduces cilia in the otic placode. Staining as in (**a**). Scale bar: 25 µm. **e** Quantification of otic placode cilia in 3 and 5 embryos injected with CTRL MO or Wdr4 ATG MO, respectively. ***p = 0.0003, Welch’s *t* test. Mean SEM. Scale bar: 10 µm. **f** Visualization of cilia in the neural tube by detection of acetylated tubulin (magenta). Shown are typical sections of CTRL MO or WDR4 ATG morphants. Scale bar: 10 µm. Wdr4 KD reduces primary (**g**) and motile (**h**) cilia. **g** *n* = 6/5, **p = 0.0043, Mann–Whitney test; **h** *n* = 6/5, ****p < 0.0001, Welch’s *t* test. **i** Wdr4 KD affects cilia in the pronephros. Staining as in (**a**). Scale bar: 10 µm. **j** Pronephric cilia are shortened upon loss of Wdr4. *n* = 6 with 170 and 193 cilia, ****p < 0.0001, Mann–Whitney test. **k** Induction of glomerular cysts upon loss of Wdr4. Pictures show cyst-free and cystic (arrows) glomeruli using the tg(wt1b:eGFP) reporter line. Scale bar: 100 µm. **l** Quantification of glomerular cyst formation after injection of two different MOs. *n* = 3 with 69–115 embryos; CTRL MO vs. Wdr4 ATG MO ****p < 0.0001; splCTRL MO vs. Wdr4 splMO ****p < 0.0001. One-way ANOVA with Sidak correction. Red line: median.

Our results prompted us to more closely investigate cilia after Wdr4 depletion. We started with the analysis of the ciliated organ of laterality, the KV in zebrafish (Fig. 2a–c). Neither area nor cell number of the KV was altered (Fig. S4a–c). However, while we found only a subtle reduction in cilium length in the KV (Fig. 2b), we counted substantially fewer cilia per KV (Figs. 2c and S3d) suggesting a defect in ciliogenesis. To corroborate this finding, we also assessed cilia in other ciliated organs of the zebrafish. In the otic placode as well as the neural tube we counted considerably fewer cilia after Wdr4 KD (Fig. 2d–h). Moreover, cilia in the pronephric duct showed length shortening upon Wdr4 KD (Fig. 2i, j). Increased frequency of renal cysts in embryos with reduced Wdr4 suggests physiological relevance of this ciliogenesis defect (Fig. 2k, l).

**Fig. 3:**
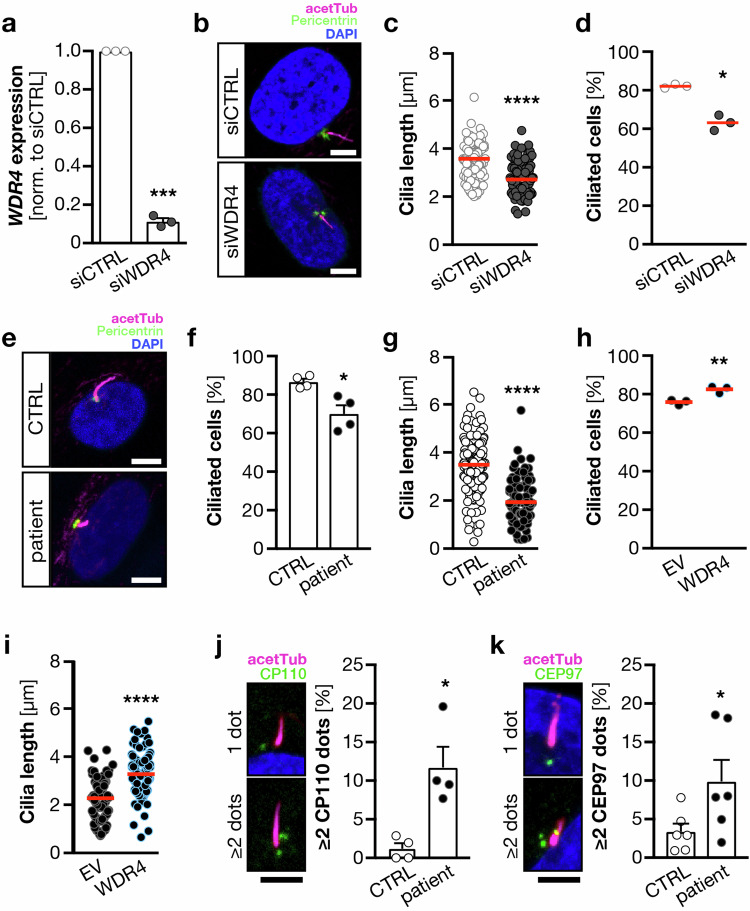
Cilia defects in the absence of WDR4 in human fibroblasts. **a** siRNA transfection of human fibroblasts results in reduced WDR4 expression. qPCR normalized to housekeeping gene (*SDHA*) and to siCTRL transfected cells. *n* = 3. ***p = 0.0003, paired *t*-test. **b** Immunofluorescence of cilia in fibroblasts transfected with control or WDR4 siRNA. Magenta: acetylated tubulin (cilia); green: pericentrin (PCNT, centrioles). Scale bar: 5 µm. **c** Cilia are shorter upon WDR4 KD. *n* = 3 experiments with 95–99 cilia in total. ****p = 0.0001. Welch’s *t* test. **d** WDR4 KD cells have fewer cilia. *n* = 3 experiments. *p = 0.0108. Unpaired, Welch’s *t* test. **e** Immunofluorescence of control and patient-derived primary fibroblasts. Staining as in (**a**). Scale bar: 5 µm. **f** Wdr4 patient fibroblasts have fewer cilia. *n* = 4 experiments. *p = 0.0255. Welch’s *t* test. **g** WDR4 patient fibroblasts have shorter cilia. *n* = 4 experiments with 108–174 cilia in total. ****p < 0.0001. Mann–Whitney test. **h** Nucleofection of WDR4 patient fibroblasts with a plasmid encoding human WDR4 rescues the percentage of ciliated cells. *n* = 3 experiments. **p = 0.01. Welch’s *t* test. **i** Reconstitution with WDR4 restores cilium length in patient fibroblasts. *n* = 3 experiments with 99 and 100 cilia. ****p < 0.0001. Welch’s *t* test. **j** Wdr4 patient cells show increased frequency of aberrant CP110 staining (green). *n* = 4 experiments with 407 and 411 cells in total. *p = 0.0455. Paired *t*-test. Mean + SEM. Scale bar: 3 µm. **k** Wdr4 patient cells show increased frequency of aberrant CEP97 staining (green). *n* = 6 experiments with 625 and 617 cells in total. *p = 0.0342. Paired *t*-test. Mean + SEM. Scale bar: 3 µm. Red line indicates median.

We turned then to human fibroblasts (Fig. 3), in which cilium formation can be induced through prolonged serum starvation. We transfected cells with a pool of four siRNAs against WDR4 or respective control siRNAs and then serum-starved for 3 days. qPCR analysis showed successful KD of WDR4 after RNA interference (Fig. 3a). Immunofluorescence analysis of these cells revealed shortened cilia (Fig. 3b, c) and a significant reduction of cells extending cilia in general (Fig. 3d). Overexpression of zebrafish Wdr4 fully rescued cilia shortening in siWDR4 fibroblasts and increased slightly, yet significantly the number of ciliated cells (Fig. S5). We further analyzed primary fibroblasts derived from the Seckel syndrome-like patients carrying the R84H variant, which display a markedly reduced expression of WDR4 (Fig. S3). Similar to our KD experiments, we observed both, shorter as well as fewer cilia compared to control fibroblasts (Fig. 3e–g). Nucleofection of patient fibroblasts with a plasmid encoding human WDR4 rescued the number of cells extending cilia (Fig. 3h) and fully restored cilium length (Fig. 3i). We further found that CP110 and its interaction partner CEP97 accumulated more frequently at the base of cilia in patient cells than in control cells, suggesting a cilium assembly defect (Fig. 3j, k). Several studies have demonstrated that both proteins need to be removed from the mother centriole to allow for cilium extension. When CP110 and CEP97 persist at the mother centriole, cilium formation is impeded [34, 35]. Could therefore be a centrosome defect the underlying cause for altered cilium formation? To test this, we treated fibroblasts and zebrafish embryos with ciliobrevin D (CBD), which has been shown to deplete cells of functional cilia [36]. CBD treatment reduced ciliation of fibroblasts also in our hands, yet did not affect centrioles as judged by γ-Tubulin staining (Fig. S6). Treatment of zebrafish embryos with CBD, induced highly similar phenotypes as WDR4 KD, e.g., small heads, small eyes, and otolith defects (Fig. S6). Yet, combination of WDR4 KD with CBD treatment did not significantly worsen this phenotype. This suggests that loss of WDR4 seems to trigger its effect mostly through cilia defects and less by causing centrosome defects. To further test a possible impact of WDR4 on centrioles, we analyzed spindle poles in mitotic HEK 293T cells after KD of WDR4 and did not detect changes as compared to controls (Fig. S7). This again suggests that centrioles remain largely unaffected by loss of WDR4.

In order to rule out that the differences between control and patient cells are due to changes in the cell cycle or leaky arrest in G0, we analyzed cyclin A2 levels in arrested cells by immunofluorescence and Western blotting (Fig. S8). Both approaches did not reveal any differences in G0-arrested cells. However, patient cells showed a non-significant delay in cell cycle entry upon release, which did not translate into a slowed cell cycle, since control and patient cells grow with similar kinetics (Fig. S8). Moreover, cilium resorption occurred at the same pace as in control cells (Fig. S8). Hence, dysfunction or loss of WDR4 likely prevents normal cilium extension.

WDR4 also could affect cilia via disturbed actin organization, since it activates the small GTPase RAC1 through degradation of Arhgap17 [32]. To test this, we analyzed stress fibers in control and patient fibroblasts. As we did not detect a difference, we consider actin organization to be largely unaffected by WDR4 loss in our hands (Fig. S9).

**Fig. 4:**
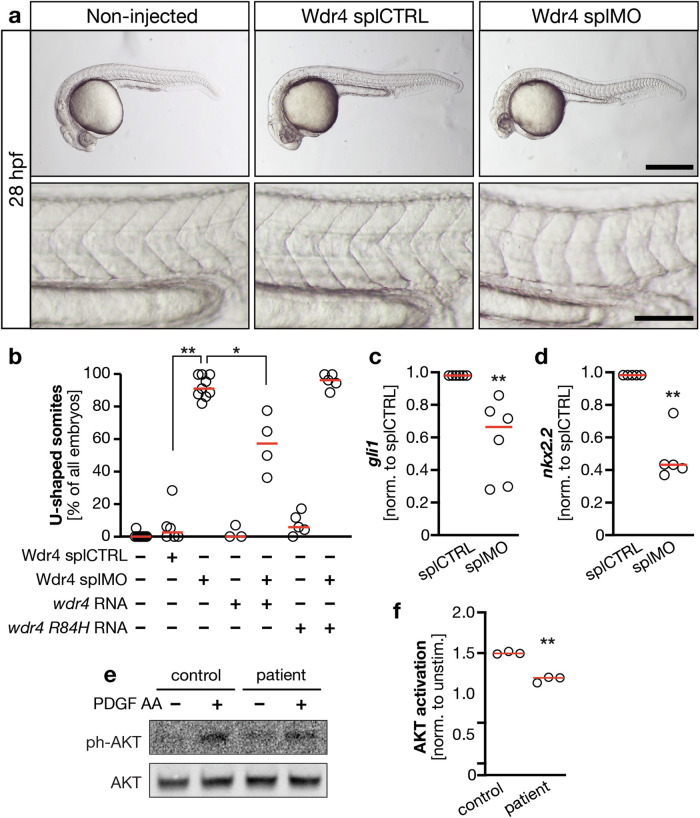
Loss of WDR4 dampens ciliary signaling capacity. **a** Embryos were left non-injected or were injected with either splCTRL MO or Wdr4 splMO and scored for U-shaped somites at the age of 28 hpf as a read-out for Hedgehog signaling. Only embryos injected with Wdr4 splMO show this defect. Lower panels show same embryos with higher magnification. Scale bars: upper panels 500 µm, lower panels 100 µm. **b** Co-injection of *wdr4* RNA partially rescues induction of U-shaped somites upon loss of Wdr4. *n* = 3–10 experiments with 103–189 embryos. splCTRL vs. splMO **p = 0.0033; splMO vs. splMO + *wdr4* RNA *p = 0.015; Kruskal–Wallis test with Dunn’s post-test. **c** Expression of *gli1* in injected zebrafish embryos. Normalized to *gapdh* and splCTRL. *n* = 6; **p = 0.0022. Mann–Whitney test. **d** Expression of *nkx2.2* in injected zebrafish embryos. Normalized to *gapdh* and splCTRL. *n* = 6; **p = 0.0079. Mann–Whitney test. **e** WDR4 patient cells show lower capacity for AKT activation upon stimulation with PDGF AA. **f** Quantification of AKT activation upon stimulation with PDGF AA. Signals were normalized to unphosphorylated AKT and nonstimulated. *n* = 3; **p = 0.0012. Welch’s *t* test. Red line: median.

Length alterations are known to affect ciliary function [37]. The occurrence of renal glomerular cysts indeed suggested dysfunctionality of cilia upon Wdr4 KD (Fig. 2k, l). To more directly assess the ability of cilia to properly serve as signaling platforms, we assessed the formation of U-shaped somites in Wdr4 morphant embryos. This defect arises, if Hedgehog (Hh) signaling is altered, a pathway that relies on functional primary cilia [38]. Loss of Wdr4 induced U-shaped somite formation, what could be partially rescued by co-injection of *wdr4* RNA, but not *wdr4 R84H* RNA (Fig. 4a, b). Furthermore, expression of Hh target genes *gli1* and *nkx2.2* was significantly reduced after Wdr4 KD (Fig. 4c, d). Additionally, WDR4 patient fibroblasts showed reduced capability to activate AKT upon stimulation with PDGF AA, a process that also depends on the cilium [39] (Fig. 4e, f). These data suggest that WDR4 enables cilium function.

**Fig. 5:**
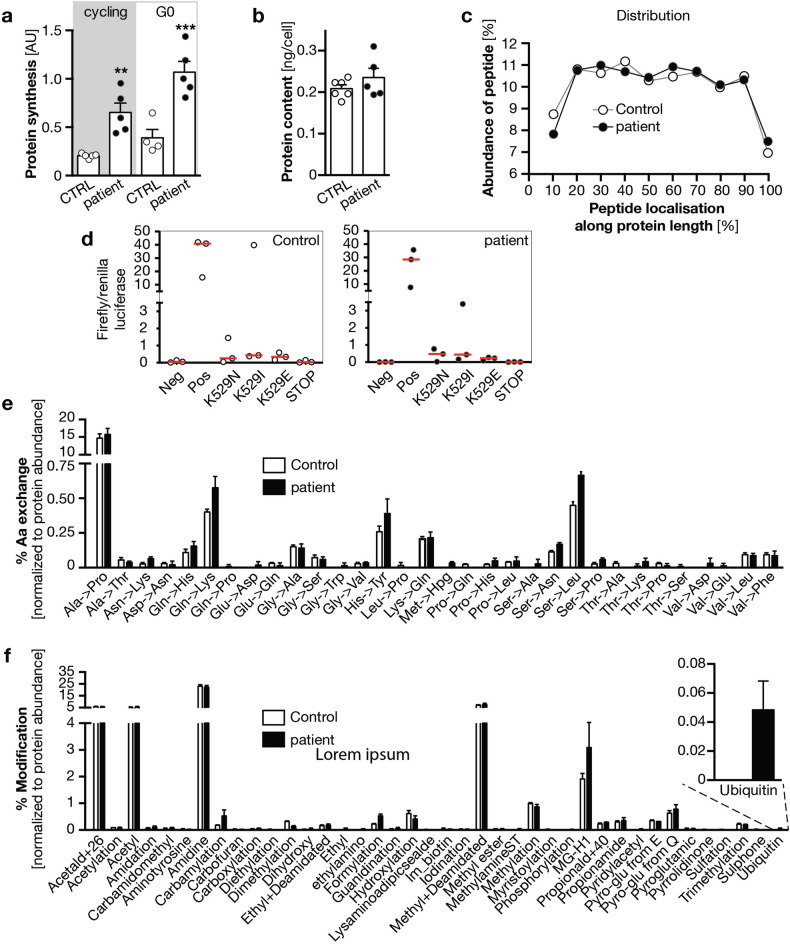
WDR4 patient fibroblasts are not deficient in protein synthesis. **a** Nascent protein synthesis is increased in WDR4 patient fibroblasts under cycling and resting conditions. *n* = 4–5. *p* = 0.0038 (**) and 0.0002 (***). Welch’s *t* test. **b** Overall protein content is not changed in WDR4 patient fibroblasts. *n* = 5–6. Mann–Whitney test. **c** WDR4 patient cells do not have more premature abortions during protein synthesis. Bar graph shows the distribution of peptides detected by mass spectrometry along the whole length of the protein. *n* = 2. **d** Assessment of translation fidelity in control and WDR4 patient fibroblasts using a luciferase based assay. Cells were transfected with firefly luciferase reporter constructs including the indicated mutations in the active center of the luciferase. Elevated luciferase activity indicates erroneous translation and reduced translational fidelity. Firefly luciferase activity was normalized to co-transfected renilla luciferase activity. **e** Bar graph showing amino acid exchange rates in control and WDR4 patient fibroblasts. *n* = 2. **f** Bar graph depicting posttranslational modifications in control and WDR4 patient fibroblasts. Inset shows ubiquitination modification. *n* = 2.

WDR4 is generally known as a co-factor for the methyltransferase METTL1, which modifies several RNAs including tRNAs. tRNA modification in turn regulates the efficacy and precision of protein synthesis [20, 40]. We hence hypothesized that loss of WDR4 in patients or KD models could potentially cause insufficient and imprecise protein synthesis. Interestingly, however, measurements of de novo protein synthesis in control and patient derived fibroblasts showed a marked increase, which was even bigger in quiescent, ciliated cells (Fig. 5a). Yet, despite the increase in synthesis, overall protein content was not changed between control and patient-derived fibroblasts (Fig. 5b). Moreover, mass spectrometry analysis demonstrated that proteins were synthesized until the very C-terminal end suggesting that the translation machinery was still able to produce regular full-length proteins (Fig. 5c). A luciferase-based assay further demonstrated that WDR4 patient fibroblasts exhibit normal translation and folding fidelity (Fig. 5d). In line with this, we also did not find a higher frequency of amino acid exchanges using mass spectrometry (Fig. 5e) and concluded that despite increased translation, proteins were still synthesized largely without errors. We next applied an algorithm on our spectrometry data to predict post-translational protein modifications. This analysis revealed a substantial increase in protein ubiquitination in patient cells carrying the WDR4 R84H variant (Fig. 5f).

Because of these unanticipated findings, we questioned the importance of the functional interaction of WDR4 with METTL1 regarding the ciliopathy-like phenotypes in fish and cells. We hence knocked down METTL1 in fibroblasts. A pool of siRNAs against METTL1 almost completely depleted all *METTL1* RNA, while also producing a very moderate reduction of *WDR4* (Fig. S10). Previous experiments showed that KD of WDR4 with such low efficacies does not impact cilia length or number (data not shown). Importantly, efficient METTL1 KD did neither affect ciliation nor cilia length (Fig. S10). These results suggest that WDR4 influences ciliogenesis likely independently of METTL1.

To better understand the underlying mechanism, we analyzed transcriptome changes induced by the WDR4-R84H variant via RNAseq. We identified 3431 differentially expressed genes (DEGs, Fig. S11a), which associated with transcriptional signatures of cell types present specifically in the central nervous system [41] (Fig. S11b). We also detected DEGs known to be connected to cilia [42] as well as DEGs involved in ubiquitin-associated processes [43], yet both sets of genes were not enriched in the group of DEGs (Fig. S11c, d). However, since differential expression of genes with ubiquitin-association seemed more pronounced (Fig. S11e), we focused our attention on ubiquitin-related processes.

**Fig. 6:**
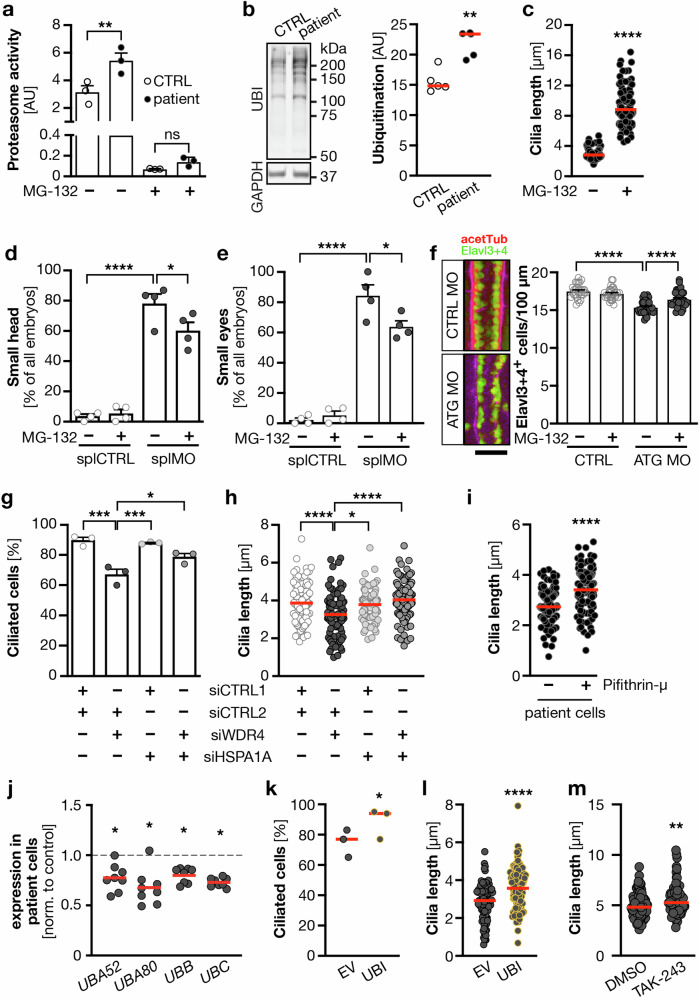
WDR4 patient fibroblasts display increased proteasomal activity. **a** Proteasome activity is increased in WDR4 patient fibroblasts compared to control cells. Specificity of the assay is shown by complete signal abrogation by treatment with the proteasome inhibitor MG-132 (25 µM). *n* = 3 experiments. **p < 0.01. One-way ANOVA with Sidak’s post-test. **b** WDR4 patient cells display higher ubiquitination. Western blot for ubiquitin and GAPDH as loading control. **p = 0.0079. Mann–Whitney test. **c** Proteasome inhibition rescues cilia length in WDR4 patient fibroblasts. *n* = 3 experiments with 91–98 cilia in total. ****p < 0.0001. Mann–Whitney test. **d** Proteasome inhibition partially rescues the microcephaly in zebrafish embryos at 48 hpf. *n* = 4 experiments with 76–116 embryos in total. *p < 0.05, ****p < 0.0001. One-way ANOVA with Sidak’s post-test. **e** Treatment with a proteasome inhibitor also rescues the small eye phenotype in 48 hpf zebrafish. *n* = 4 experiments with 76–116 embryos in total. *p < 0.05, ****p < 0.0001. One-way ANOVA with Sidak’s post-test. **f** Wdr4 KD reduces the number of neuronal cells (Elavl3 + 4, green) in the neural tube of 24 hpf zebrafish, which can be rescued by proteasomal inhibition. Counterstaining for acetylated tubulin (magenta). *n* = 3 experiments with 45 embryos in each condition. ****p < 0.0001, Kruskal–Wallis test with Dunn’s post-test. Scale bar: 50 µm. **g** Simultaneous knockdown of HSPA1A partially rescues ciliation defect in human fibroblasts. *n* = 3, siCTRL1/siCTRL2 vs. siWDR4/siCTRL2 ***p = 0.0004; siWDR4/siCTRL2 vs. siHSPA1A/siCTRL2 ***p = 0.0007; siWDR4/siCTRL2 vs. siWDR4/siHSPA1A *p = 0.0255. One-way ANOVA with Sidak correction. **h** Simultaneous knockdown of HSPA1A rescues cilia length in human fibroblasts. *n* = 3, 96–104 cilia, siCTRL1/siCTRL2 vs. siWDR4/siCTRL2 ****p < 0.0001; siWDR4/siCTRL2 vs. siHSPA1A/siCTRL2 *p = 0.0119; siWDR4/siCTRL2 vs. siWDR4/siHSPA1A ****p < 0.0001; Kruskal–Wallis test with Dunn’s post-test. **i** The HSPA1A inhibitor pifithrin-µ restores cilium length in WDR4 patient cells. Fibroblasts were treated with 5 µM pifithrin-µ during the whole time of serum starvation. *n* = 3 with 103 (CTRL) and 112 (pifithrin-µ) cilia in total. ****p < 0.0001. Two-tailed Welch’s test. **j** qPCR analyses shows reduced expression of ubiquitin precursors in serum-starved patient fibroblasts. *p = 0.0156 (*UBA52*, *UBB*, *UBC*) and 0.0312 (*UBA80*). Wilcoxon tests. **k** Ciliation rate can be rescued in patient cells nucleofected with a ubiquitin plasmid. *n* = 3 experiments. Per experiment at least 100 cells were counted. *p = 0.0246. Paired *t*-test. **l** Cilium length in WDR4 patient fibroblasts can be rescued by nucleofection of a ubiquitin plasmid; *n* = 3 with 105 cilia each, ****p < 0.0001. Welch’s *t* test. **m** Cilium length in WDR4 patient fibroblasts can be rescued by inhibition of ubiquitin activating enzymes with 5 nM TAK-243; *n* = 3 with 96 and 99 cilia. **p < 0.0062. Mann–Whitney test. Red line: median.

Protein modification by covalent addition of single or multiple ubiquitin molecules serves many different purposes in the cell. The most commonly known ubiquitin modification is polyubiquitination of proteins, which targets them for proteasomal degradation. This marks dysfunctional proteins for removal, yet also to recycle the components for new synthesis including supplying the cell with free ubiquitin again [44]. Consistent with the increased appearance of ubiquitin modifications we detected an increased proteasomal activity in WDR4 patient fibroblasts in G0 (Fig. 6a). Addition of the proteasome inhibitor MG-132 completely abolished proteasomal activity in control and in patient cells (Fig. 6a). Western blot analysis of the same cells further demonstrated an increase in protein ubiquitination (Fig. 6b). We then tested, whether inhibition of the proteasome has an influence on cilia in patient cells and observed increased cilium length upon treatment with MG-132 (Fig. 6c). Furthermore, MG-132 treatment of zebrafish embryos was in itself sufficient to at least partially rescue the size of head and eyes in zebrafish (Fig. 6d, e). MG-132 also increased the number of neuronal cells in the neural tube, which were reduced upon Wdr4 KD (Fig. 6f). These data suggest that uncontrolled proteasomal degradation of proteins and subsequent cilium extension deficits due to WDR4 loss-of-function could potentially reduce the number of neuronal cells during embryogenesis and in turn predispose to microcephaly.

In order to find an explanation for the increased proteasomal activity we turned to our mass spectrometry analysis. HSPA1A was the most abundant protein in patient cells and 5-fold upregulated compared to control cells (Table S1). Interestingly, high levels of HSPA1A have been reported to stimulate the proteasomal activity [45]. Therefore, we performed double KD of WDR4 and HSPA1A in human fibroblasts. KD efficiency could be verified and neither smartpool altered the expression of the other transcript (Fig. S12a, b). While depletion of HSPA1A alone affected neither ciliation nor cilium length, we observed a complete rescue of cilium formation and cilium length in WDR4 siRNA cells co-transfected with siRNAs against HSPA1A (Fig. 6g, h). In addition, pharmacological inhibition of HSPA1A using the small molecule pifithrin-µ [46] similarly restored cilium length in WDR4 patient cells (Fig. 6i). We further hypothesized that the production of ubiquitin precursors should be upregulated to render the increased proteasomal activity possible. Contrary to that we detected a reduction of all four ubiquitination precursors using qPCR on control and WDR4 patient fibroblasts after serum starvation (Fig. 6j). Yet, nucleofection of patient cells with a construct encoding for free ubiquitin, which cannot be conjugated [47] increased the percentage of cells extending cilia and these cilia were significantly longer (Fig. 6k, i). Additionally, we suppressed ubiquitination in WDR4 patient fibroblasts by treatment with TAK-243, an inhibitor of ubiquitin activating enzymes. This was sufficient to trigger a subtle, yet significant rescue of cilia length (Fig. 6m). Together, these data suggest that loss of WDR4 may lead to a shortage of free ubiquitin, which the cell attempts to counter-regulate by increased proteasomal activity.

## Discussion

WDR4 has originally been identified as a co-factor of METTL1 in the modification of tRNAs at position 46 [40]. We initially hypothesized the WDR4-METTL1 complex to be essential for protein synthesis as its dysfunction in yeast led to the degradation of tRNAs [48]. One explanation for impaired brain and head growth in WDR4 patients and KD zebrafish would thus be a failure in overall or selected protein synthesis. This idea was further bolstered by a report about another methyltransferase (NSUN2), which is implicated in microcephaly and associated with decreased translation of mRNAs into protein [49]. Furthermore, deregulation of protein synthesis has been postulated to be causative for other neurodevelopmental disorders, e.g., Rett syndrome [50]. WDR4 patient fibroblasts, however, did not display any impairment in protein synthesis, instead de novo translation was elevated. Moreover, the increase in de novo protein synthesis did not yield a higher protein content, which may be the consequence of the observed higher proteasomal activity. Our subsequent hypothesis that loss of WDR4 may lead to errors in protein sequences and would hence activate the proteasome to clear out the faulty proteins could neither be confirmed. WDR4 patient cells possess a normal translation and folding fidelity. These observations suggest that the cilia defects upon WDR4 loss-of-function are not necessarily due to a general lack in proteins or accumulation of dysfunctional proteins. Intriguingly, though, inhibition of the proteasome by MG-132 rescued cilium length as well as partially the microcephaly and neurogenesis defect in zebrafish. The proteasome can impact on cilia in multiple ways, such as prevention as well as promotion of ciliogenesis, control of axoneme extension and cilium resorption (reviewed in [51]). Additionally, MG-132 has been used to prevent degradation of specific factors that affect ciliogenesis such as trichoplein [52] or applied to globally affect proteostasis to rescue cilium length in contexts such as autophagy-defective cells [53]. Interestingly, MG-132 enriches mostly newly synthesized proteins, which characteristically are short-lived such as signaling and membrane proteins on which the cilium depends greatly [54]. Hence, the cilium defect in WDR4 cells could possibly be caused by a shortage of certain cilia components. We do not have experimental evidence to confirm or reject this hypothesis, but similar observations have been made for cilium defects resulting from decreased mTOR signaling, which was suggested to provide a specific subset of proteins required for ciliogenesis [55].

Our study is fortified by data obtained in mice, where WDR4 has previously been connected to very specific ubiquitination events. Here, WDR4 was instrumental to mark Pml [27] and Arhgap17 [32] for ubiquitin-mediated degradation, the latter being necessary for proper development of the murine cerebellum [32]. Using patient-derived cells, we identify a much further reaching impact of WDR4 on protein synthesis, ubiquitination and hence protein homeostasis. We propose that heatshock proteins as well as ubiquitin pools potentially drive this process. High levels of the heatshock protein HSPA1A, which is upregulated in WDR4 patient cells, provoke increased proteasomal activity [45]. Protein degradation by the proteasome, on the other hand, has been shown to generate free ubiquitin [56]. These data together with our observation that ubiquitination is elevated in WDR4 patient cells renders the question whether WDR4 loss-of-function cells lack sufficient amounts of free ubiquitin. Reduced expression of ubiquitin precursors also points in this direction. Sufficiently high ubiquitin levels are important during development. Lack of UBC results in embryonic lethality at midgestation [57] and deletion of UBB impairs neural differentiation [58]. Moreover, free ubiquitin levels appear to be particularly high in the brain [59]. Indeed, supplementation with free ubiquitin did rescue the cilium defect in cells (Fig. 6h, i).

Cilia are at the heart of development and have previously also been connected to PM [60]. WDR4 dysfunction does not only impair cilium formation in many cells and organs, but impairs also their functionality as we detected dampened activity of different signaling pathways that rely on the cilium. The Hh pathway may be the most important in this context, since it is crucial for brain development. Yet, previous studies failed to detect changes in Hh pathway activity after loss of WDR4 [32]. This may be explained by the lack of agonist stimulation as this is necessary to induce measurable Hh signaling changes in cell culture. The symptoms observed in patients carrying WDR4 variants, however, most likely are due to an interplay of more than one mechanism. The above-mentioned impact of WDR4 on Arhgap17 degradation is an important example of a contributing factor [32] and it would be interesting to assess cilia in the presence of accumulated Arhgap17. Additionally, METTL1 as well as WDR4 propagates the expansion of neuronal cells and the differentiation of neuronal cells from iPSC cells [20] suggesting an additional impact on normal brain development by the canonical METTL1-WDR4 complex. There may also be a centrosomal contribution as in other PM cases. This, however, will be subject of subsequent studies.

Taken together, we here report that WDR4 is indispensable for proper cilium generation. This provides a new axis along which WDR4 contributes to faithful brain and skull development and helps to better understand how WDR4 variants contribute to PM.

## Materials and methods

### Materials

The following, already established cell lines were obtained from the University of Sussex’s cell line collection at the Genome Damage and Stability Centre: h-Tert immortalized 1BR3 forearm fibroblasts, h-Tert immortalized WDR4 patient-derived fibroblasts, non-immortalized WDR4 patient-derived fibroblasts and lymphoblastoid cell lines from WDR4 patients (c.251G > A; p.R84H). HEK 293T cells were from the American Type Culture Collection. MG-132 and TAK-243 were purchased from Selleckchem, pifithrin-µ from Sigma-Aldrich and ciliobrevin D from MedChemExpress. Antibody sources are indicated in the respective methods sections.

### Zebrafish maintenance and manipulation

Zebrafish were maintained in a ZebTec rack system with automated water recycling and adjustment of water parameters based on automated pH, conductivity and temperature measurements (Tecniplast). Fish were maintained under a 14 h light and 10 h dark cycle and fed three to four times a day with living artemia in the morning (Aqua Schwarz) and pelleted food at least twice thereafter (Zebrafeed, Sparos). Used lines in here were AB and EK wild-type lines as well as a reporter line expressing GFP in the glomerulus and proximal pronephros. WDR4 loss-of-function was achieved by microinjection of antisense morpholino oligonucleotides (MO) into the yolk of 1–2 cell stage embryos for ubiquitous targeting. MOs were designed and synthesized based on submitted sequences by Gene Tools Inc (Oregon, USA) and had the following sequences: Wdr4 splMO: 5’-CCCACCTGCAAGAGGACAATCATAC, Wdr4 splCTRL: 5’-CACACCTCCAACAGGAAAATAATAC, Wdr4 ATG MO: 5’-CACTACAGCCATGTTGAGTGTTGTT. In addition, a standard control MO provided by GeneTools was used. For reconstitution experiments, capped RNA encoding different variants of zebrafish Wdr4 (which resembled human wild-type and patient variants) was co-injected with MO. In all experiments, uninjected wild-type controls as well as embryos injected with a non-targeting control MO (Wdr4 splCTRL or the standard control MO provided by GeneTools) of the same clutch of eggs were included to assess clutch quality and to identify injection-based off-target effects. Eggs of the same clutch were distributed randomly into the different treatment groups. Embryos were raised at 28.5 °C and incubated to the desired stages. No blinding occurred as the phenotypes were too obvious to ensure blinding during analyses. Splice blocking was verified by RT-PCR as follows: Total RNA was isolated from 24 hpf embryos using the Quick-RNA Miniprep kit (Zymo Research), equal amounts were transcribed into cDNA using Superscript III reverse transcriptase (Thermo Fisher) and oligodTTPs (IDT). PCR was performed using Tag polymerase (NEB) and the following primers: Forward: 5’- GGA GGG ATC AGA AGA GAA GGA, Reverse: 5’- GCC TGA ATG TTG TAG GGT GAA. In case the MO inhibits splicing, exon 4 should be excised and a smaller PCR band occurs. Treatment with 2.5 µM ciliobrevin D was from tailbud stage on.

### Analysis of cartilage formation

Zebrafish embryos were fixed at 4 days post fertilization (dpf) using 4% buffered paraformaldehyde (PFA). After fixation, embryos were shortly rinsed with PBS and transferred first to 50% EtOH in PBS for 10 min and then to staining solution (0.02% Alcian blue, 70% EtOH, 50 mM MgCl_2_). After overnight incubation at room temperature zebrafish were once rinsed with H_2_O and then bleached for 20 min (bleaching solution: 1.5% H_2_O_2_ in 1% KOH) and gradually cleared (30 min 20% Glycerol/0.25% KOH, 2 h 50% Glycerol/0.1% KOH). Stained embryos were stored at 4 °C in 50% Glycerol/0.1% KOH.

### In situ hybridization

Whole mount *i*n situ hybridization (ISH) was performed according to standard protocols using DIG-labeled antisense probes, an alkaline phosphatase-coupled anti-DIG antibody (Roche) and NBT/BCIP (Roche) for color development. A probe was generated to detect *wdr4* by NotI linearization of the plasmid described below and in vitro transcription using SP6 RNA polymerase (NEB) along with Roche’s DIG labeling system. All other probes used have been described previously [61].

### Cloning

For generation of an antisense probe against zebrafish *wdr4* a 986 bp fragment of NM_001126435 was amplified using the Expand long template PCR System (Roche) from cDNA of 24 hours post fertilization (hpf) zebrafish embryos. The PCR was cleaned up with the Qiagen nucleotide removal system and cloned into pCRII by TOPO TA cloning (Life technologies). For generation of capped RNA zebrafish Wdr4 (NM_001126435.2) was cloned via BamHI and EcoRI into pCS2-GFP resulting in a C-terminal fusion of GFP to Wdr4. Site-directed mutagenesis was used to generate the patient-resembling pCS2-GFP-Wdr4-R84H. Capped RNA was synthesized using Ambion’s SP6 mMessage mMachine Kit after linearization of the template plasmid using NotI. A plasmid containing human WDR4 was obtained from Origene. Site-directed mutagenesis was used to insert the R84H mutation.

### Cell culture and transfection

hTert-immortalized fibroblasts were cultured in MEM alpha containing essential amino acids, penicillin/streptomycin and 10% fetal calf serum (FCS) (all Thermo Fisher) at 37 °C and 5% CO_2_. Primary cells were cultured just the same except for 15% FCS in the medium. Lymphoblastoid cells were cultured in RPMI 1640 medium (Thermo Fisher) supplemented with 15% FCS, penicillin, and streptomycin. siRNA-mediated transfections were carried out using pools of siRNAs (either Dharmacon’s SmartPools or siRNA pools from siTools) and Lipofectamine RNAiMAX (Thermo Fisher) according to the manufacturer’s protocol. Nucleofections were carried out with the AMAXA nucleofector IIb and the AMAXA Nucleofector Kit R (both Lonza). Cilium formation was induced by serum deprivation (0.1% FCS) for 72 h. Cells were regularly monitored for mycoplasma contamination and authenticated using highly polymorphic short tandem repeat loci sequencing. A plasmid carrying GFP-tagged zebrafish Wdr4 was used for rescue experiments.

### qPCR

RNA was isolated using Zymo’s Quick-RNA MiniPrep system, which includes genomic DNA removal and an on-column DNase digest. cDNA was transcribed with equal amounts of total RNA using either Thermo Fisher’s Super Script II or NEB’s Protoscript II kit, in both cases using oligodTTP for priming and following the manufacturer’s protocol. qPCR was performed with Roche’s Universal Probe System, Luna Universal Probe qPCR Master Mix (NEB) and a LightCycler 480 (Roche) or Quantstudio 5 (Thermo Fisher Scientific). qPCR assays were designed with the help of the Universal Probe Assay Design Center (Roche). Primers and corresponding probes are given in Supplementary Table 1.

### Protein synthesis assay

Newly synthesized protein amounts were measured with the help of the Click-iT® Plus OPP Protein Synthesis Assay Kits (cat. no. C10456, Molecular Probes) according to the manufacturer’s instructions.

### Proteasome activity assay

Proteasome activity in human wild-type and patient-derived fibroblasts was analyzed with the Proteasome 20S Activity Assay Kit (cat. no. MAK172, Sigma).

### Translation fidelity

Translation fidelity was measured essentially as described before ([62] and references therein). 10^6^ cells were nucleofected using the Amaxa system (Lonza), using 5 µg firefly control/mutant reporter plasmid and 0.1 µg renilla luciferase plasmid. Cells were plated in a 96-well plate (5 × 10^4^ cells/well in 75 µl) and allowed to grow for 24 h. Luciferase activity was measured using Dual-Glo assay (Promega). The ratio of firefly to renilla luciferase activity was calculated and used as an indicator of translational fidelity.

### Measurement of protein content

1BR3 fibroblasts and primary WDR4-derived fibroblasts were cultured in a 10 cm dish. When cells reached confluency, cells were trypsinized, counted using a Countess II (Thermo Fisher) and 350,000 cells each pelleted in an Eppendorf tube. Cells were washed once with pre-warmed PBS, pelleted again and all liquid was carefully aspirated. Cells were lysed then in SDS lysis buffer (see also Western blotting) and protein content relative to a bovine serum albumin calibration curve was measured with the help of a bicinchoninic acid (BCA) kit (Sigma-Aldrich).

### Sample preparation and mass spectrometry (MS) analysis

Cell pellets for whole proteome analysis were lysed using 7 M Urea, 2 M Thiourea, 30 mM Tris-HCl, pH 8.5. After 1:6 dilution, proteins were reduced, alkylated and subsequently digested overnight using a trypsin to protein ratio of 1:50. The resulting peptides were lyophilized, reconstituted in 15 µl 5% TFA and analyzed via HPLC-MS. Both, chromatography and MS analysis was performed as described previously [63], using an LTQ Orbitrap Velos Pro system (Thermo Fisher Scientific). Data have been deposited at MassIVE (Id: MSV000092847).

### MS data analysis and statistics

For peptide distribution analysis, database search was performed using MaxQuant version 1.6.3.4 (www.maxquant.org) [64]. Employing the build-in Andromeda search engine [65], MS/MS spectra were correlated with the UniProt human reference proteome set (www.uniprot.org) for peptide identification. Carbamidomethylated cysteine was considered as a fixed modification along with oxidation (M), and acetylated protein N-termini as variable modifications. False Discovery rates were set on both, peptide and protein level, to 0.01.

Data analysis was repeated using PEAKS Studio (BSI Inc., Version 8.5) employing a database search with similar parameters and conducting a subsequent SPIDER search to allow for AA exchange and PTM discovery. For normalization, peptide counts were divided by the total peptide count in the respective sample.

### RNA sequencing and bioinformatical analysis

Total RNA was isolated with the Quick-RNA MicroPrep system (Zymo) after cilia induction through serum starvation. Sequencing of RNA samples was performed using Illumina’s next-generation sequencing methodology [66]. In detail, total RNA was quantified and quality checked using Tapestation 4200 in combination with RNA ScreenTape (both Agilent Technologies). RIN was >9.5 for all samples (average 9.9). Libraries were prepared from 200 ng of input material (total RNA) using NEBNext Ultra II Directional RNA Library Preparation Kit in combination with NEBNext Poly(A) mRNA Magnetic Isolation Module and NEBNext Multiplex Oligos for Illumina (Unique Dual Index UMI Adaptors RNA) following the manufacturer’s instructions (New England Biolabs). Quantification and quality checked of libraries was done using an Tapestation 4200 and a D5000 assay (Agilent Technologies). Libraries were pooled and sequenced on a NovaSeq 6000. System runs in SP v1.5/101 cycle/single-end/standard loading workflow mode. Using 19 cycles in index 1 read, the index as well as the UMI sequences were obtained. Sequence information was converted to FASTQ format using bcl2fastq v2.20.0.422. Sequencing approach resulted in 58–75 m total reads per sample (av. 66 m).

Mapping of reads to the reference was done using TopHat v2.1.0 [67] using parameters --no-convert-bam --no-coverage-search -x 1 -g 1. As reference the human Ensemble genome version GRCh38 was used taking the gene annotation Release 92 into account [68]. Depending on the sample 77–83% of the reads could be mapped uniquely to the reference. PCR duplicons occurred in library preparation were removed after mapping of reads based on UMIs (unique molecular identifier) using umi_tools v1.1.1 [69]. Around 25–60% of reads per sample passed de-duplication process. Remaining reads per gene were counted for each sample using featureCounts v2.0.3 [70] using parameter -s2. Around 90% of reads per sample could be assigned for counting. Gene counts were further processed using the programming language R. In order to find differentially expressed genes (DEG) the statistical packageDESeq2 [71] was applied. *p* values were adjusted for multiple testing according to Benjamini–Hochberg correction (FDR). Genes with adjusted *p* value < 0.05 were regarded to be differentially expressed. For further analysis, changes of 20% in expression were regarded as relevant. Metascape (v3.5.20230501) [41], Syscilia [42], and Biogrid [43] platforms were used to analyze data and identify genes associated with cilia and ubiquitin-related processes, respectively.

### Western blotting and PDGF AA assay

Fibroblasts were seeded at a density of 60,000 cells per well in a 12-well plate. On the next day, full medium was removed and cells were exposed to starvation medium for the following 72 h. Cells were then washed once in pre-warmed PBS and lysed in 60 µl SDS lysis buffer (2% SDS and 50 mM Tris pH 6.8) containing protease and phosphatase inhibitors (Roche, Germany). In case of PDGF AA assays, cells were treated with DMSO as control or PDGF AA (50 ng/ml, BioLegend) during 3 min before lysis. Lysates were cleared by nuclease treatment (Pierce universal nuclease, Thermo Fisher) and centrifugation. Protein content was assessed by BCA (Sigma-Aldrich) and equal amounts of protein were separated on Bolt Bis-Tris 4–12% gradient gels using MES running buffer (both Thermo Fisher) before blotting onto nitrocellulose membranes (Bio-Rad). Blots were blocked in 3% milk in Tris buffered saline containing 0.1% NP-40 (Applichem) and subsequently incubated in primary antibody over night at 4 °C. After three washes with Tris buffered saline containing 0.1% NP-40, secondary antibodies were applied for 2 h at room temperature or at 4 °C overnight. Signals were detected using a Li-COR Odyssey SA system and the preinstalled Li-COR software or alternatively with an iBright FL1500 and iBright Analysis Software (version 5.2.0, both Thermo Fisher). Chemiluminescence was used to detect signals in Westerns shown in Fig. S3a. Primary antibodies used were: mouse anti-GAPDH (Acris antibodies, clone 6C5, 1:1000 or Millipore cat. no. MAB6374, 1:500), mouse anti-phospho-AKT S473 (Rockland, cat. no. 200-301-268, 1:1000), rabbit anti-AKT (Cell Signaling, clone C67E7, cat. no. 4691, 1:1000), mouse anti-ubiquitinylated proteins (Merck Millipore, clone FK2, cat. No. 04-263, 1:1000), rabbit anti-WDR4 (Santa Cruz, cat. no. sc-83348, 1:500), rabbit anti cyclin A2 (Proteintech, cat. no. 18202-1-AP, 1:500), and mouse-anti-γTub (Sigma,1:1000) Secondary antibodies for Li-COR were IR-dye labeled and used at 1:10,000 dilution (Li-COR) or for iBright FL1500 Alexa Fluor plus 800 or 647 labeled (Thermo Fisher). Uncropped images of membranes are shown in Fig. S13.

### Immunofluorescence and cilium analysis

Fibroblasts grown on cover slips were fixed either with 4% PFA at room temperature for 10 min or using ice-cold methanol for a duration of 4 min. Before antibody incubation, cells underwent a 10 min permeabilization step using 0.1% Triton X-100 in PBS and 1 h of blocking in PBS containing 10% (FCS) or 5% bovine serum albumin Fraction V. Primary antibodies were diluted in blocking buffer and applied to cells over night at 4 °C. After three washes, secondary antibodies diluted in blocking buffer were applied for 2 h at room temperature. After three more washes with PBS, coverslips were mounted onto glass slides using Vectashield mounting medium containing Dapi (Vector labs) and edges sealed with nail polish.

Zebrafish embryos were fixed over night at 4 °C in 4% PFA and stored in absolute methanol at −20 °C. Cilia were stained according to standard protocols [72]. In order to visualize cilia in the Kupffer’s vesicle the tail portion of the embryos was separated from the rest of the embryo and the majority of yolk was gently removed using forceps. This deyolked portion of the embryo was then flat-mounted in a small drop of Vectashield containing Dapi between two cover slips sealed with vacuum grease.

Antibodies used were: rabbit anti-CEP97 (Sigma-Aldrich, catalog no. HPA002980, 1:200), rabbit anti-CP110 (Sigma-Aldrich, cat. no. HPA039402, 1:100), rabbit anti-Elav3 + 4 (GeneTex, cat. no. GTX128365, 1:1000), rabbit-anti-pericentrin (Sigma-Aldrich, cat. no. HPA019887, 1:1000), rabbit anti-PKCζ (Santa Cruz Biotechnology, cat. no. sc-216, 1:500), rabbit anti cyclin A2 (Proteintech, cat. no. 18202-1-AP, 1:500), rabbit anti-centrin 1 (Proteintech, cat.no. 12794-1-AP, 1:300), mouse anti-acetylated tubulin (Sigma-Aldrich, clone 6-11B-1, 1:1000), mouse anti-γTubulin (Sigma-Aldrich, clone GTU-88, 1:5000), rabbit anti-γTubulin (Sigma-Aldrich, T6793, 1:5000), mouse anti-alpha1-tubulin-FITC (Sigma, F2168, 1:500). Unlabeled primary antibodies were detected with Alexa-coupled secondary antibodies (Thermo Fisher). CoraLite594-Phalloidin (Proteintech) was used to detect stress fibers.

### Imaging

Live zebrafish embryos and such processed by whole mount ISH were imaged on a Leica M125 with an IC80HD or a MC190HD camera (Leica, Wetzlar, Germany). For the analysis of smaller heads, eyes and shorter bodies, injected embryos were compared to average non-injected and control-injected controls. KV cilia in flat-mounted tails of zebrafish embryos as well as primary cilia in human fibroblasts were imaged using a Leica TCS SP5II confocal microscope or Leica Stellaris 5. Here, z-stacks were captured every 0.3–4 µm and processed into a 3D stack with the Leica acquisition software LAS AF and LAS X, respectively. All image processing was done using Adobe Photoshop CC2020 (Adobe Systems Software Ireland Limited, Dublin, Ireland). Adjustments (brightness, contrast or color balance) were applied equally to the whole image and equally to same treatment groups (i.e., CTRL MO vs. Wdr4 MO). Final figures were assembled in Adobe Illustrator CC2021.

### Cilium and neuronal cell measurements

To measure cilia length, cilia were traced in z-stacks and the length measured in FIJI (NIH). Neural tubes of 24 hpf zebrafish embryos were imaged on a M205FCA stereomicroscope equipped with a DFC9000 GT sCMOS camera and the LAS X software (all Leica). Elav3 + 4-positive cell bodies were counted along a 300 µm stretch of the neural tube.

### Statistical analysis

Data were analyzed using Prism 8, 9, and 10 respectively (GraphPad). Data were first assessed for normal distribution with a Shapiro–Wilks test, before the respective two-tailed parametric or non-parametric test was applied. Whenever more than two conditions were compared, an ANOVA-based test was applied. Transformation was applied to make data samples obtained as percentage amenable for analysis by ANOVA [73].

## Supplementary information

Supplemental Material

## Data Availability

The datasets generated during the current study are available from the corresponding author upon reasonable request. Datasets of transcriptome analysis have been deposited in NCBI’s Gene Expression Omnibus [74] and are accessible through GEO Series accession number GSE242969. Datasets of proteome analysis of the study have been deposited in the MassIVE repository with the identifier MSV000092847 (https://massive.ucsd.edu/ProteoSAFe/private-dataset.jsp?task=9c0bb17c77249dfb4cb2bfc5afd2c7a).
