# Supplementary material for: Cilia defects upon loss of WDR4 are linked to proteasomal hyperactivity and ubiquitin shortage
Source: Cell Death Dis. 2024 Sep 9;15(9):660. doi: 10.1038/s41419-024-07042-5 (PMC11384789; doi:10.1038/s41419-024-07042-5)
Supplement: Supplementary file 1 — Supplemental Material [file 41419_2024_7042_MOESM1_ESM.pdf]

Supplementary information: Burkhalter et al.

Protein	Fold change [patient/CTRL]
HSPA1A	5.114979835
HSPA6	3.910370327
HSPH1	5.4258112
HMOX1	2.915604935
S100A6	2.605917895
DDB1	2.149418329
LAMB2	4.242036312
HSPE1	2.130354151
RPS15	4.762071341
NPM3	3.6629251
SMC2	3.562207935
CKAP4	-3.026063557
RPL7	-4.168488802
RAP1B;RAP1A	-3.844916032
RPL13A	-5.472793139
S100A4	-2.896251109
RPL6	-6.274136108
COL1A1	-3.769591103
COL1A2	-5.534413011
COL12A1	-2.713459265
RPL4	-2.696146932
NT5E	-3.980746781
RPS11	-4.082976019
LRRC47	-4.058592764
ARL1	-4.445871808

Supplementary information; Table S1: Significantly changed proteins in WDR4 patient fibroblasts compared to control fibroblasts as measured by mass spectrometry.

Gene	Forward primer (5'-)	Reverse primer (5'-)	Universal Probe
HSPA1A	cggaaggaccgagctctt	ggctccgctctgagattg	47
METTL1	agagagctcaggcccaagt	cagcggtgacagttccacta	49
SDHA	ggacctggttgtctttggtc	ccagcgtttggtttaattgg	80
UBA52	tccggttccgctatcttct	ggtcttcacaaagatctgcatgt	70
UBA80	cctgatcagcagagactgatctt	ttttcttagcaccaccacga	11
UBB	aggatcctggtatccgctaac	tcacattttcgatggtgtcact	39
UBC	ggaaggcattcctcctgat	cccacctctgagacggagta	11
WDR4	gtgttcttcgtgggaactgg	ggccttgtagagactgctgaa	11
gapdh	caggcataatggttaaagttggta	catgtaatcaaggtcaatgaatgg	147
gli1	ggtctcgatgccagtgga	cactgacggagccagtcc	5
nkx2.2a	actagatggctcgcaaccac	cttgagagttcgcggacag	89

Supplementary information; Table S2: Primers and probes employed in qPCR assays

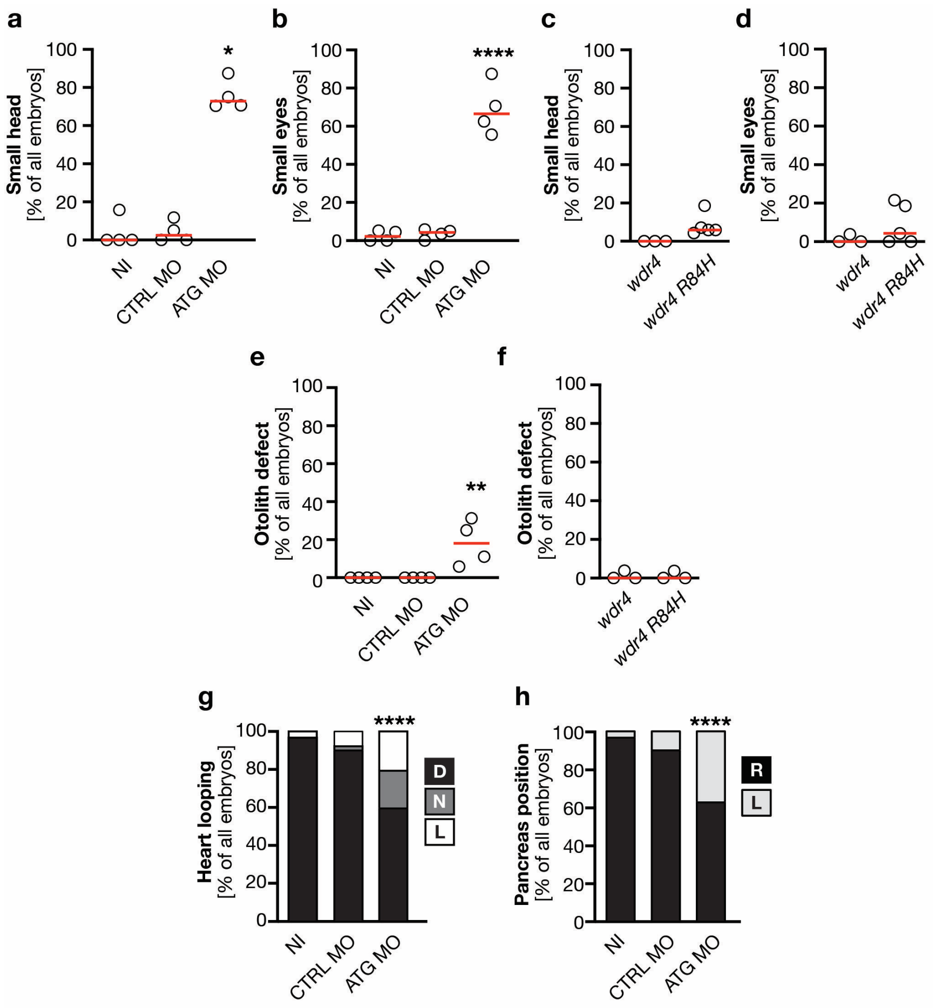

Supplementary information; Fig. S1: Translation blocking morpholino (ATG MO) targeting expression of *wdr4* replicates defects observed after injection of splMO, while injection of RNA encoding *wdr4* or *wdr4-R85H* on its own has no effect.

a, Increased frequency of embryos showing a reduced head size upon injection of the ATG translation blocking WDR4 MO. n=4 experiments with 92-105 embryos, CTRL MO vs ATG MO, * p=0.0194, Kruskal-Wallis test with Dunn's post test.

b, Increased frequency of embryos showing smaller eyes upon injection of the ATG WDR4 MO. n=4 experiments with 92-105 embryos, CTRL MO vs ATG MO, **** p<0.0001, one-way ANOVA with Sidak correction.

c, d, Injection of capped RNA encoding *wdr4* or *wdr4*-R85H does not induce small heads (c) or eyes (d). n=4 experiments with 92-105 embryos

e, Increased frequency of otolith defects upon injection of the ATG WDR4 MO. n=4 experiments with 92-105 embryos, CTRL MO vs ATG MO, ** p=0.0051, Kruskal-Wallis test with Dunn's post test.

f, Injection of capped RNA encoding *wdr4* or *wdr4*-R85H does not induce otolith defects.

g, ATG MO increases wrong heart looping. n=3, CTRL MO vs ATG MO; correct vs. wrong looping. n=90 and 106; **** p<0.0001, Fisher's exact test.

h, ATG MO randomizes pancreas placement. n=3, CTRL MO vs ATG MO, n=90 and 107; **** p<0.0001, Fisher's exact test.

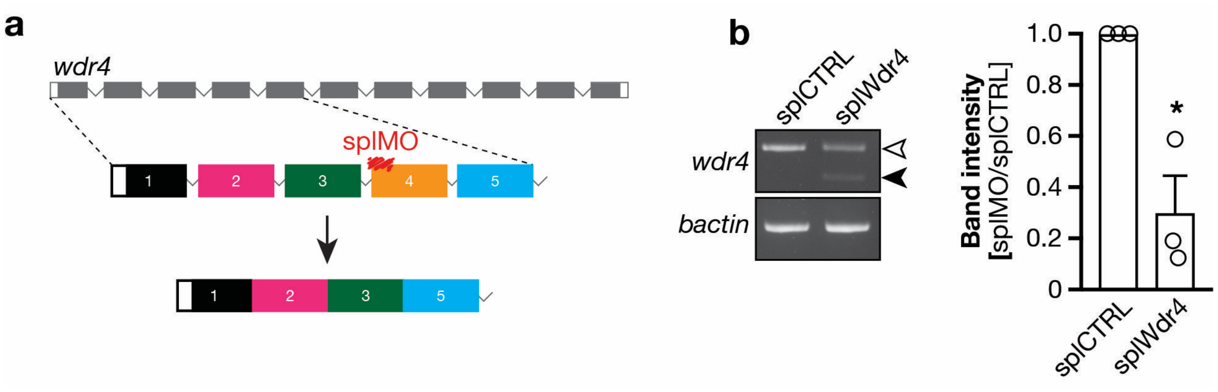

Supplementary information; Fig. S2: Splice blocking verification of Wdr4 splice MO

a, Schematic representation of how the splice blocking MO affects *wdr4* splicing. Grey boxes indicate all exons of wdr4, below are the first 5 exons enlarged and represented as coloured boxes. Binding of the splMO at the 5'-end of exon 4 and consequent loss of exon 4 is indicated.

b, RT-PCR of 24 hpf zebrafish embryos, which were injected with a Wdr4 splice control MO (splCTRL) and a Wdr4 splice MO (splMO), respectively. Regular Splicing was inhibited as can be seen by the additional, lower band in the Wdr4 splMO lane. Additionally, decrease in intensity of the upper band in the Wdr4 splMO lane was quantified. Bars indicate mean and SEM. Circles indicate individual experiments. n=3, * p=0.0402, paired t-test.

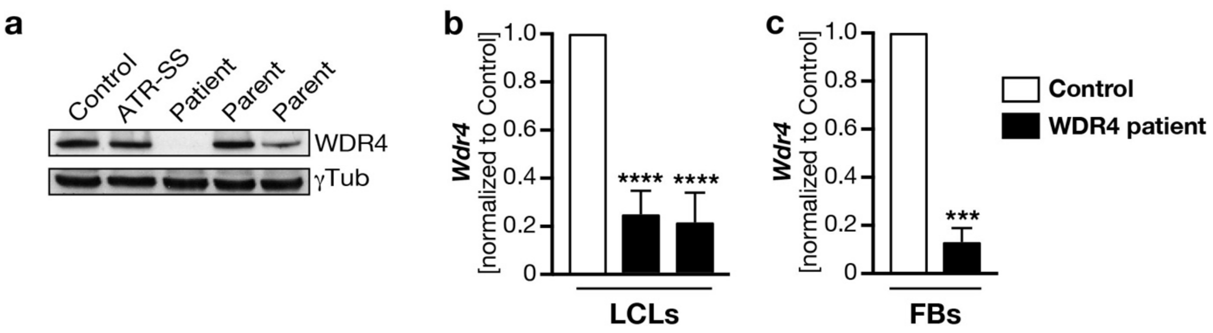

Supplementary information; Fig. S3: WDR4 expression is reduced in cells derived from microcephalic children carrying the R84H variant

a, Western blot of lymphoblastoid cell lines (LCLs) from different donors including the parents of a patient carrying the R84H variant for WDR4. As control for microcephaly syndromes a lysate from ATR-SS, ATR Seckel Syndrome patient-derived LCLs was used. γTubulin serves as loading control.

b, qPCR of control and two patient LCLs showing a reduction of *WDR4* transcripts. n=4. **** $p<0.0001$, One-way ANOVA with Dunnett's multiple comparison test.

c, qPCR of control and patient-derived fibroblasts (FBs) showing a reduction of *WDR4* transcripts. n=4. *** $p=0.0007$, paired, two-tailed t-test.

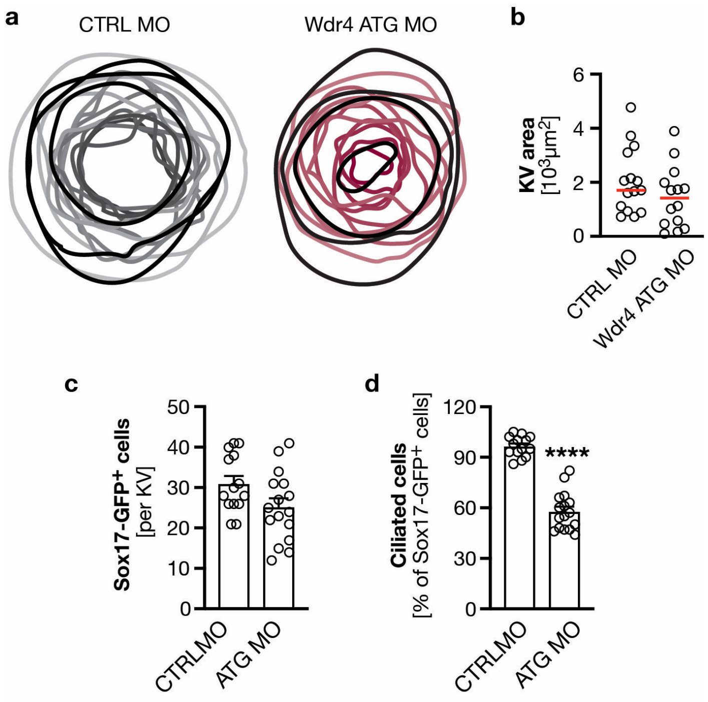

Supplementary information; Fig. S4: Lack of Wdr4 only affects ciliation, but not area or cell number in the Kupffer's vesicle.

a, Outline of KVs visualized in CTRL MO- or Wdr4 ATG MO-injected embryos (8 somite stage).

b, Area of KVs does not significantly change by loss of Wdr4 n=15/14; two-tailed Welch's test.

c, Cell number of the KV does not significantly change upon reduction of Wdr4 as judged by the number of Sox17-GFP$^+$-cells

d, Sox17-GFP$^+$-cells show reduced ciliation upon loss of Wdr4. n=2experiments with 14 and 16 embryos, respectively; **** $p<0.0001$, two-tailed Welch's t test.

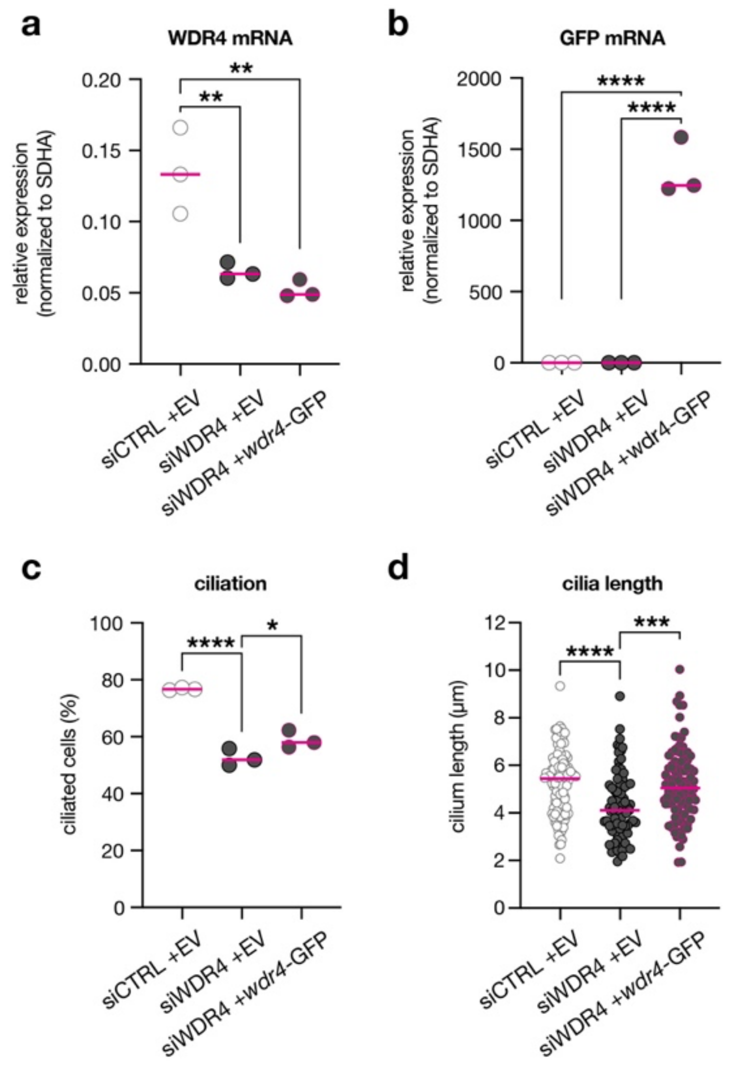

Supplementary information, Fig. S5: Overexpression of zebrafish Wdr4 rescues shortening of cilia in siWDR4 fibroblasts.

a, Transfection of siWDR4 reduces WDR4 mRNA. n=3; one-way ANOWA with Sidak post test; ** p=0.0065 and 0.0028.

b, Zebrafish Wdr4-GFP mRNA is expressed only when corresponding construct is transfected. GFP=specific primers were used. n=3; one-way ANOWA with Sidak post test; **** p< 0.0001.

c, Overexpression of zebrafish Wdr4 promotes slight rescue of ciliated cells in siWDR4 cells. n=3; one-way ANOWA with Sidak post test; **** p< 0.0001 and * p=0.042.

d, Overexpression of zebrafish Wdr4 rescues of cilia length in siWDR4 cells. n=3, n=114/84/102; Kruskal-Wallis test with Dunn's post test; **** p< 0.0001.

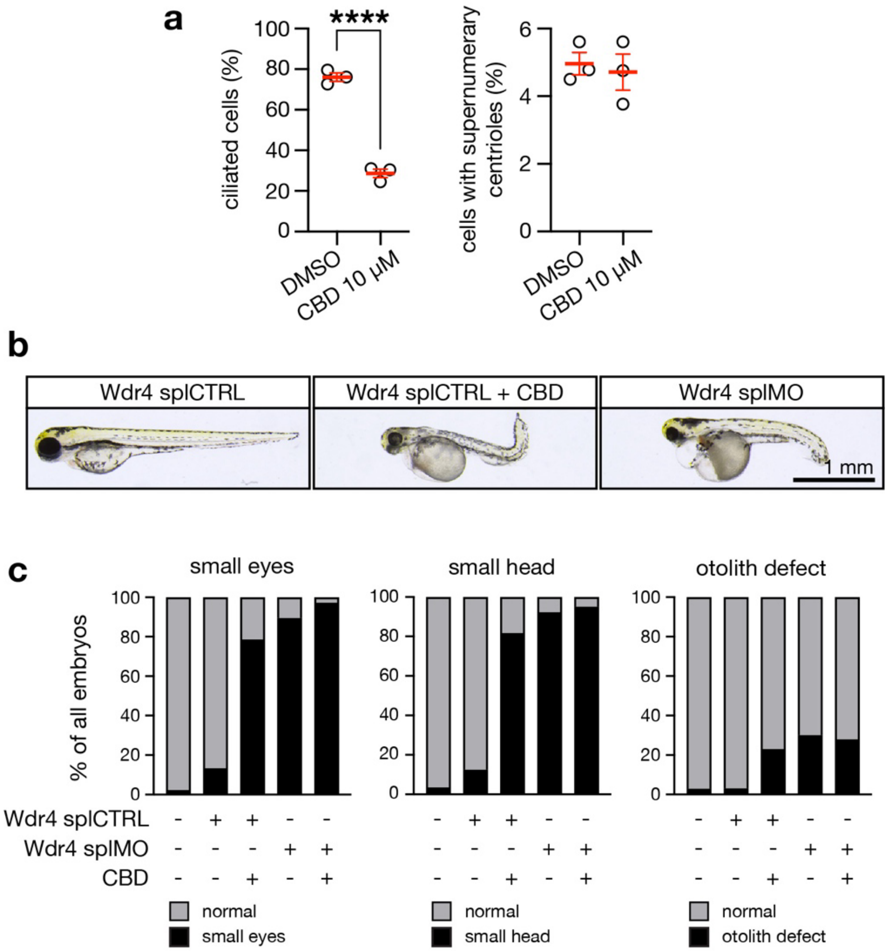

Supplementary information, Fig. S6: Treatment with ciliobrevin D does not worsen Wdr4 KD-phenotype in zebrafish embryos.

a, Ciliobrevin D treatment (10 µM, 3 days) significantly reduces ciliation in human fibroblasts (n=3, Welch's t test, **** p< 0.0001), while the frequency of supernumerary centrioles is not changed (n=3).

b, Zebrafish embryos injected with splCTRL or Wdr4 splMO and optionally treated with 2.5 µM ciliobrevin D from tailbud stage on. Ciliobrevin D treatment induces similar phenotypes to a similar extent as Wdr4 splMO.

c, Stacked bar graphs showing the percentage of embryos with small head, small eyes or otolith defects in non-injected embryos and zebrafish embryos injected with splCTRL or Wdr4 splMO and optionally treated with 2.5 µM ciliobrevin D from tailbud stage on. Ciliobrevin D does not significantly worsen the phenotype in Wdr4 morphant embryos.

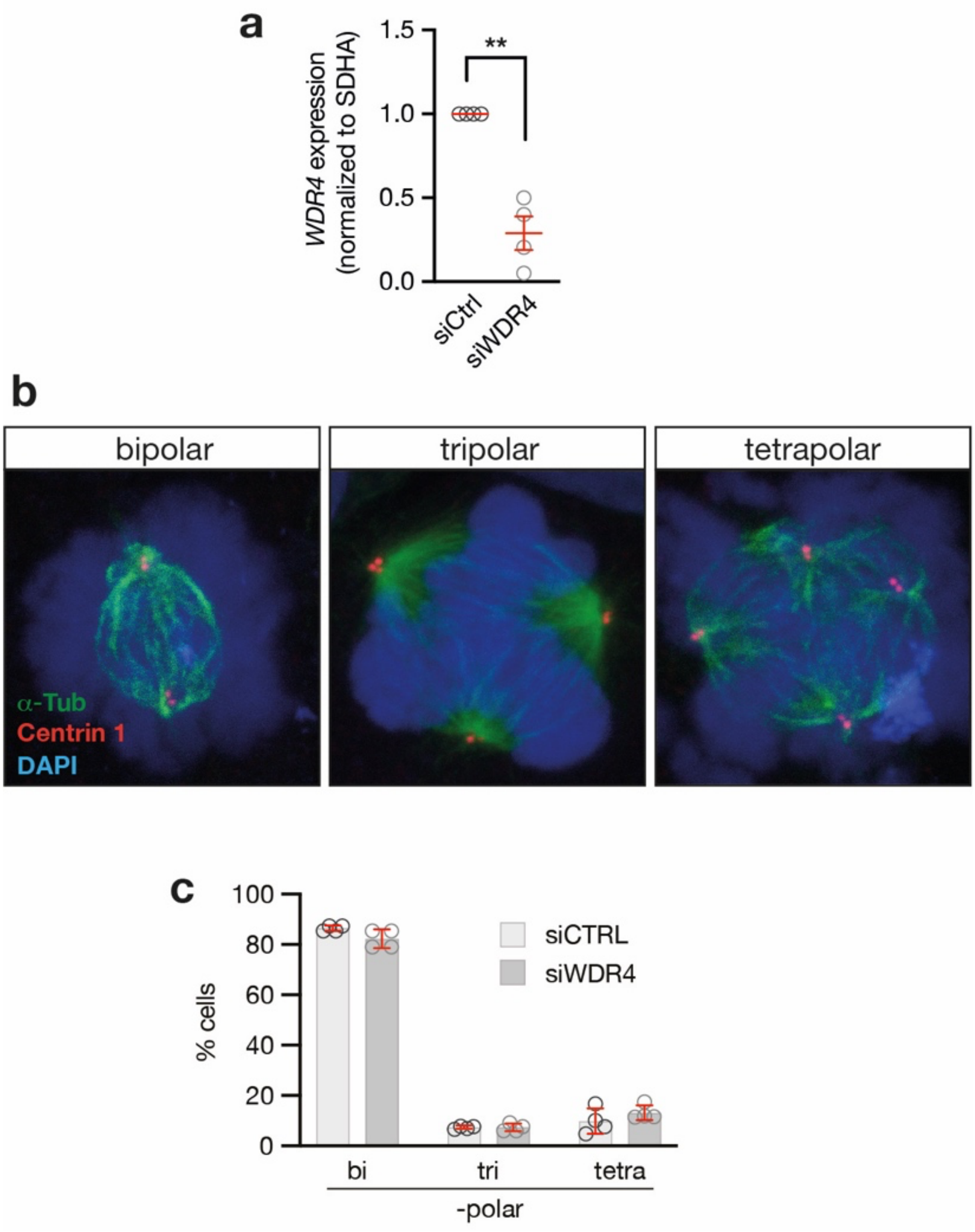

Supplementary information, Fig. S7: KD of WDR4 does not influence number of mitotic spindles.

a, Efficiency of WDR4 KD. Expression normalized to SDHA and control. n=4. One sample t and Wilcoxon test. ** p= 0.0058.

b, Examples mitotic HEK 293T cells showing bipolar, tripolar and tetrapolar spindles.

c, WDR4 KD does not alter the frequency of bi- tri-, or tetrapolar spindles. n=4. At least 200 cells were counted per experiment and condition.

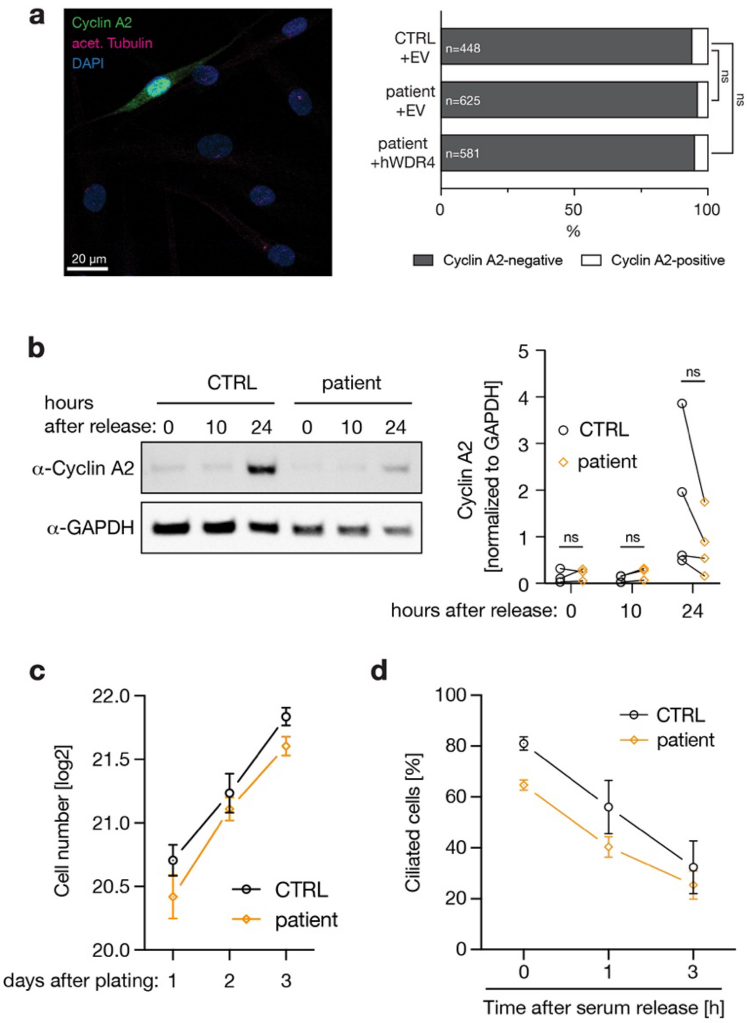

Supplementary information, Fig. S8: WDR4 patient cells show similar G0 synchronization, growth rate and cilium resorption kinetics.

a, Fibroblasts were serum starved for 72 hours and stained to allow detection of cyclin A2 as a marker for S-phase cells (green) and acetylated tubulin (magenta). No significant changes in the percentage of S phase cells in control (CTRL) and patient cells transfected with an empty vector (EV) and patient cells transfected with a vector carrying human WDR4. n=3, the number of counted cells is indicated.

b, Western blot analysis of control and patient cells that were serum starved (0) or released from starvation by addition of serum during 10 or 24 hours. Cyclin A2 was used as a marker of S phase, whereas GAPDH served as loading control. The quantification to the right indicates no difference at 0 and 10 hour time points between control and patient cells. Cyclin A shows a non-significant tendency of slower accumulation in patient cells. n=4; line between symbols connects results from individual experiments.

c, Control and patient derived cells grow with similar kinetics. n=3, Shown are means and SEM.

d, Fibroblasts were serum starved for 72 hours and subsequently normal growth medium containing serum was re-added. Cilia were counted at different time-points. While patient cells show initially a reduced rate of ciliation, an acceleration in cilium resorption cannot be observed. n=3 experiments. At least 100 cells per replicate were counted. Shown are means and standard deviation.

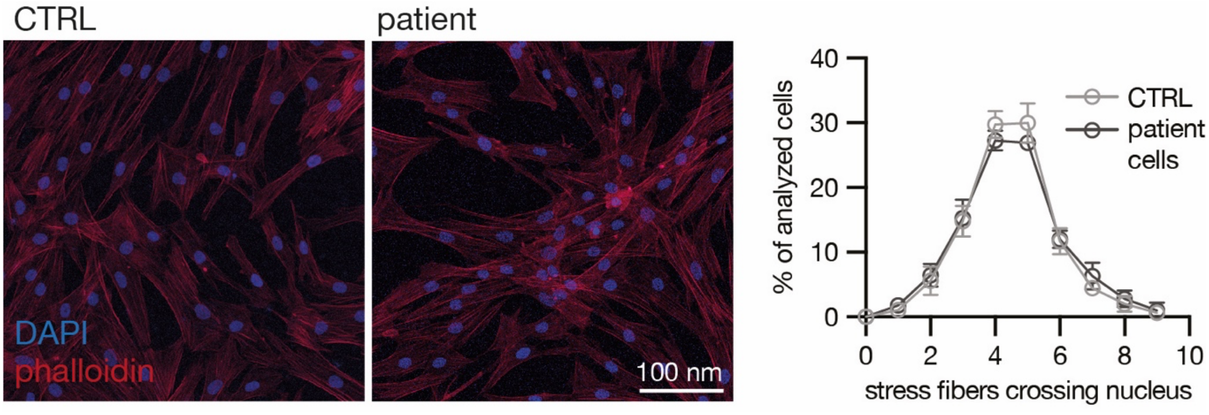

Supplementary information, Fig. S9: WDR4 KD does not alter stress fibres number.

In order to assess alterations of actin filaments in dependence of WDR4, phalloidin-CoraLite594 was used to stain stress fibres in control (CTRL) and patient cells. Loss of WDR4 did not generally alter actin filaments as judged by the number of stress fibres crossing nuclei. n=3 experiments with, 491 and 446 cells counted in total.

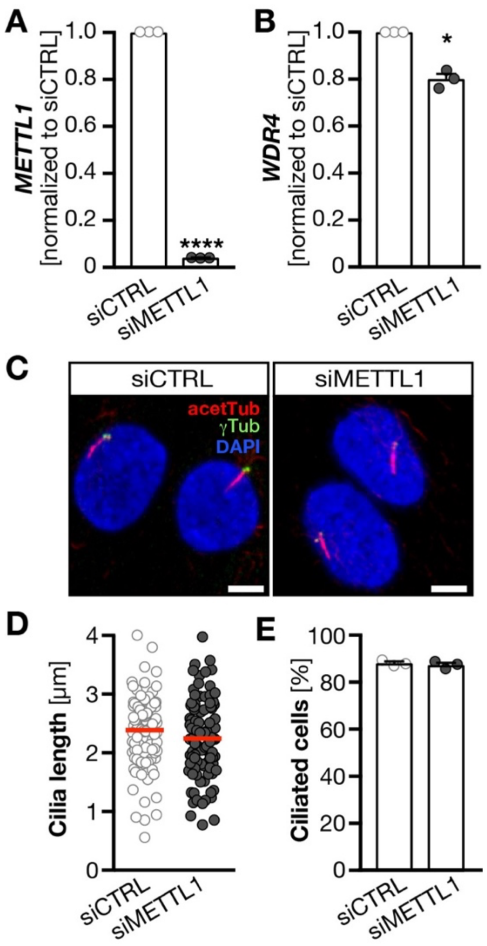

Supplementary information; Fig. S10: The ciliogenesis defects in WDR4 patient cells are unlikely caused by WDR4's functioning together with METTL1.

a, METTL1 was knocked down in human fibroblasts using a siRNA smartpool. n=3. Two-tailed paired t-test. **** p<0.0001.

b, METTLs1 knockdown affects only mildly WDR4 expression. n=3. Two-tailed paired t-test. * p=0.0118.

c, Confocal images of cilia in control and METTL1 siRNA transfected human fibroblasts. Cilia are stained using an acetylated tubulin antibody (red, acetTub) and centrioles including the basal body are counterstained for γtubulin (green, γTub). Scale bar: 5 µm.

d, Cilia length is not altered upon METTL1 knockdown. n=3 experiments with 102-109 cilia in total. Two-tailed, unpaired t-test. p=0.2553.

e, Cilia are formed at the same rate in cells depleted of METTL1. n=3 experiments. Per experiment at least 100 cells were counted. Two-tailed, unpaired t-test. p=0.5734.

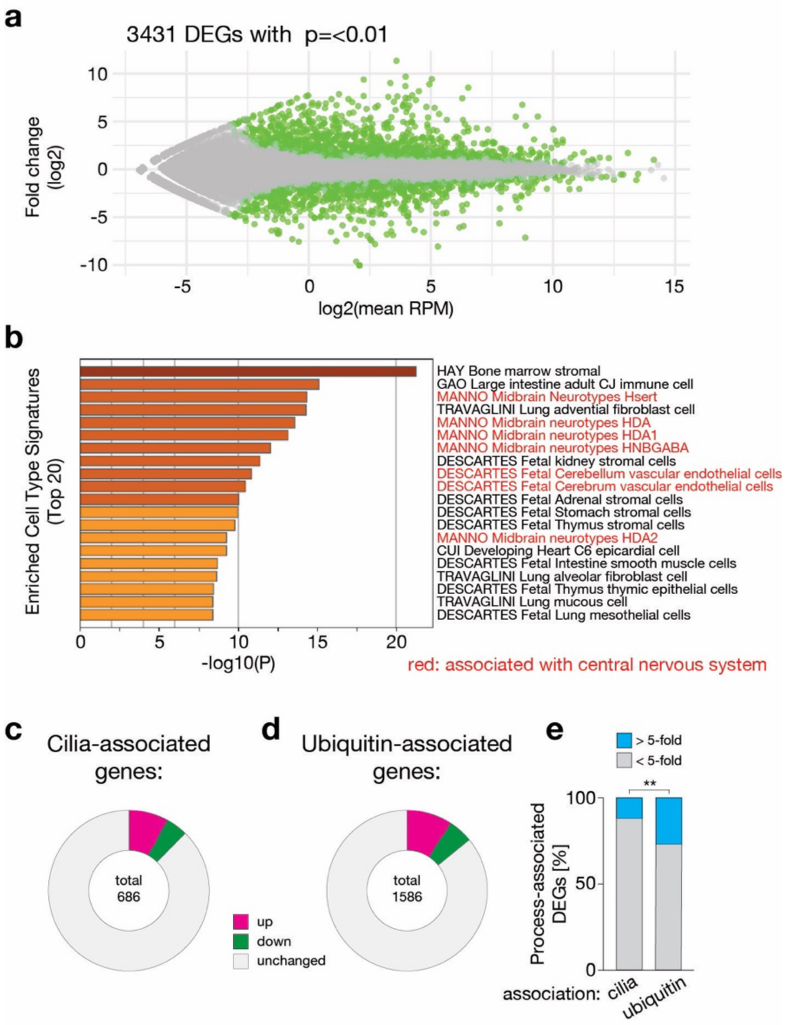

Supplementary information, Fig. S11: Transcriptome analysis of Wdr4 patient cells.

a, MA plot summarizing transcriptome analysis of WDR4 patient cells compared to control cells. 3431 differentially expressed genes (DEGs) with an adjusted p=<0.01 were identified.

b, Metascape-Analysis identified several RNA profiles in DEGs typical for midbrain cells and brain vascular endothelial cells.

c, Of 686 genes known to be associated with cilia (SCGSv2), 85 are found in 3431 DEGs (55 up- and 30 down-regulated). Enrichment of cilia-associated genes in the group of DEGs could not be confirmed; p=0.9987, Fisher's exact test.

d, Of 1586 genes known to be associated with ubiquitin-related processes (BioGRID), 224 are found in 3431 DEGs (146 up- and 78 down-regulated). Enrichment of ubiquitin-associated genes in the group of DEGs could not be confirmed; p=0.9953, Fisher's exact test.

e, Ubiquitin-associated DEGs show higher tendency to be strongly affected (26.56%; 34 of 128) as compared to cilia-associated genes (15.96%; 10 of 85). Arbitrary cut-off at 5-fold regulation, ** p=0.0096, Fisher's exact test.

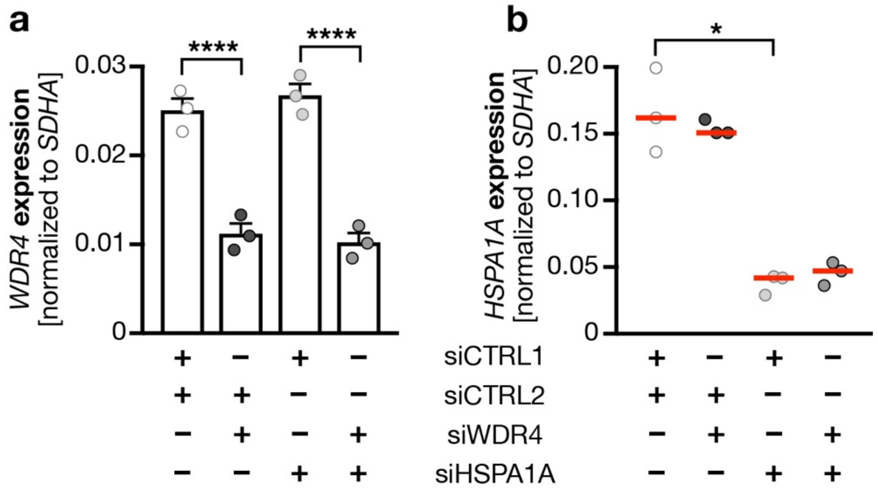

Supplementary information; Fig. S12: Simultaneous KD or inhibition of HSPA1A rescues cilia length in human fibroblasts lacking WDR4.

a, Expression of *WDR4* after siRNA transfection into control fibroblasts. n=3. One-way ANOVA. **** p<0.0001.

b, Expression of *HSPA1A* after siRNA transfection into control fibroblasts. n=3. Kruskal-Wallis test with Dunn's multiple comparison. * p=0.0255. The red line indicates the median.

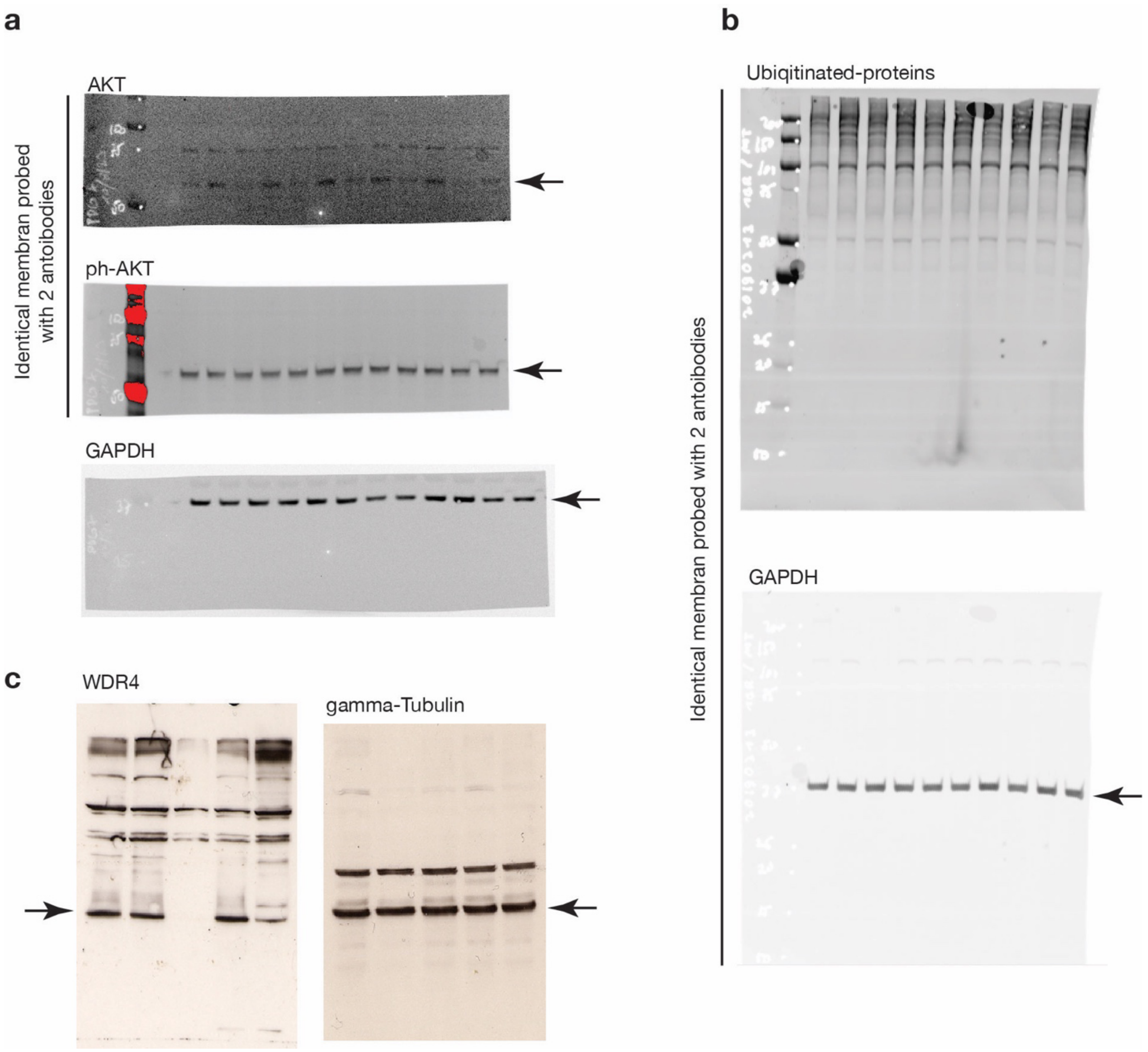

Supplementary information; Fig. S13: Uncropped Western blots.
